# Plasma Levels of sRAGE, Loss of Aeration and Weaning Failure in ICU Patients: A Prospective Observational Multicenter Study

**DOI:** 10.1371/journal.pone.0064083

**Published:** 2013-05-27

**Authors:** Matthieu Jabaudon, Sébastien Perbet, Bruno Pereira, Alexis Soummer, Laurence Roszyk, Renaud Guérin, Emmanuel Futier, Qin Lu, Jean-Etienne Bazin, Vincent Sapin, Jean-Jacques Rouby, Jean-Michel Constantin

**Affiliations:** 1 Intensive Care Unit, Department of Anesthesiology and Critical Care Medicine, Estaing Hospital, CHU Clermont-Ferrand, Université d’Auvergne Clermont Ferrand 1, Clermont-Ferrand, France; 2 R2D2– EA 7281, School of Medicine, Université d’Auvergne Clermont Ferrand 1, Clermont-Ferrand, France; 3 Department of Clinical Research and Innovation (DRCI), CHU Clermont-Ferrand, Clermont-Ferrand, France; 4 Intensive Care Unit Pierre Viars, Department of Anesthesiology and Critical Care Medicine, La Pitié-Salpêtrière Hospital, Assistance Publique-Hôpitaux de Paris, UPMC Univ Paris 06, Paris, France; 5 Department of Medical Biochemistry and Molecular Biology, Estaing University Hospital, CHU Clermont-Ferrand, Université d’Auvergne Clermont Ferrand 1, Clermont-Ferrand, France; Università Vita-Salute San Raffaele, Italy

## Abstract

**Rationale:**

Postextubation distress after a successful spontaneous breathing trial (SBT) is associated with increased morbidity and mortality. Lung ultrasound determination of changes in lung aeration predicts weaning failure. It remains unknown whether this derecruitment is related to alveolar epithelial dysfunction or not.

**Objective:**

To verify whether lung alveolar type I epithelial cell injury marker sRAGE (soluble form of the receptor for advanced glycation end-products) is predictive of postextubation distress and weaning failure or not, and to verify whether plasma sRAGE levels can be related to lung derecruitment during the process of weaning from mechanical ventilation or not.

**Interventions, Measurements:**

88 patients from 2 intensive care units were included in this observational prospective study. Plasma sRAGE levels were measured in duplicate by ELISA before, at the end of a 60-minute SBT, and 4 hours after extubation. To quantify lung aeration, a lung ultrasound score was calculated.

**Main Results:**

34% of extubated patients experienced postextubation distress. Patients with or without postextubation distress had comparable sRAGE levels before SBT, after SBT, and 4 hours after extubation. In patients with postextubation distress, sRAGE levels were not predictive of the need for mechanical ventilation. sRAGE levels were not associated with lung aeration as assessed by echography. Patients who succeeded SBT (86%) and those who failed (14%) had no differences in sRAGE levels, before (median 1111 vs 1021 pg/mL, p = 0,87) and at the end of SBT (1165 vs 1038 pg/mL, p = 0.74).

**Conclusions:**

Plasma levels of sRAGE do not predict postextubation distress or SBT failure/success in patients weaning from mechanical ventilation. Lung aeration loss during a successful weaning trial predicts postextubation distress, but may not be evaluable by plasma levels of sRAGE, a marker of alveolar type I epithelial cell injury.

**Trial Registration:**

ClinicalTrials.gov NCT01098773

## Introduction

Weaning from mechanical ventilation is a critical period in intensive care unit (ICU) patients [1], [2]. Weaning failure includes initial spontaneous breathing trial (SBT) failure, postextubation distress and death occuring within 48 h following extubation [1]. Postextubation distress is defined as reintubation or need for non-invasive ventilation within 48 hours following extubation [1], [3]. Following a successful SBT, incidence of reintubation ranges between 3 and 30% [1], [3]. Postextubation distress after a successful SBT is associated with increased morbidity and mortality [4]. Given the risks associated with delayed or unsuccessful extubation, determining readiness for extubation and predicting postextubation distress is a critical challenge in the ICU. Most of proposed predictors of postextubation distress either require special equipment, or are too complex for bedside use, or have a limited predictive value [3]. To date, there are no simple clinical indices known to be powerful predictors of postextubation distress. Many mechanisms may impact on the ability to wean from mechanical ventilation, including spontaneous breathing-induced cardiac failure, and neuromuscular disorders, or alteration of lung resistance and compliance.

Based on our recent findings, a 60-minute SBT is associated with significant lung derecruitment, as assessed by transthoracic lung ultrasound, and among patients who successfully pass SBT, the derecruitment is greater in patients who develop postextubation distress than in those who do not [5]. Factors leading to such a derecruitment have been poorly investigated to date. Among them, alveolar epithelial dysfunction, its repair, and their putative roles in maintaining lung homeostasis could be rather novel and unexplored candidates [6], [7]. In particular, a critical property of the alveolar epithelial barrier for maintaining lung fluid balance is the capacity to remove alveolar fluid by vectorial ion transport, a role shared by both alveolar type I and type II epithelial cells (6). As recently shown by our group and other teams, the soluble form of the receptor for advanced glycation end-products (sRAGE) is a marker of alveolar type I epithelial cell injury, and levels of sRAGE, are elevated during acute respiratory distress syndrome (ARDS) [8]–[10]. They are also inversely correlated with the rate of alveolar fluid clearance in an ex vivo isolated perfused human lung model, and sRAGE might therefore represent a gross estimate for alveolar type I epithelial cell dysfunction beyond ARDS (16).

The association between SBT-induced loss of aeration and alveolar type I epithelial cell injury has never been investigated to date, and it remains unknown whether plasma levels of sRAGE could be useful in identifying patients at risk for postextubation distress during the process of weaning from mechanical ventilation.

Therefore, the objectives of this study were to determine whether plasma levels of sRAGE are associated with 1) postextubation distress, 2) SBT failure, 3) lung aeration loss during a successful weaning trial.

## Methods

The protocol for this trial and supporting STROBE checklist are available as supporting information; see Checklist S1 and Protocol S1.

### Ethics Statements

The Institutional Review Board of the University Hospital of Clermont-Ferrand, France (Comité de Protection des Personnes Sud Est VI), approved the research protocol for this ancillary study. All participants, or their next of kin, provided written consent to participate in this study. There was no deviation from the approved protocol.

### Study Patients

Clinical data and biological samples for this study were prospectively obtained from patients enrolled in a multicenter observational study of ultrasound assessment of lung aeration loss during a weaning trial and its role as a predictor of postextubation distress [5]. Consecutive patients receiving mechanical ventilation for more than 48 hours were included in the primary study when the underlying disease that had required intubation was considered by the attending physician as reversed, rendering the patient eligible to a first one-hour SBT [11]. Patients were included in the present study based on the availability of their plasma samples, in order to evaluate plasma sRAGE levels in the setting of weaning from the ventilator.

### SBT

The SBT was performed through a T-tube, as previously described (5, 11), and patients who successfully passed the SBT were extubated, followed up for 48 hours and classified in the postextubation success group or in the postextubation distress group [3].

Criteria for defining successful SBT, failure of SBT and postextubation distress were chosen based on the work from Esteban et al [11].

### LUS

Lung Ultrasound Score (LUS) was calculated as previously described [5], [12], [13], before, immediately after and 4 hours after SBT. LUS was performed by trained investigators using a 2- to 4-MHz convex probe as previously described [5], [12], [13]. In all patients from each ICU, the same investigator performed the LUS at each time point of the study. Each intercostal space of upper and lower parts of the anterior, lateral, and posterior regions of the left and right chest wall was carefully examined and four ultrasound aeration patterns were defined and scored for each region of interest: normal aeration, moderate loss of lung aeration, severe loss of lung aeration and lung consolidation. LUS score ranges between 0 and 36, with higher scores indicating more severe loss of lung aeration.

### Assay Procedures

Arterial blood samples were collected from an indwelling catheter in patients before, at the end of SBT, and 4 hours after extubation. sRAGE concentrations were measured in duplicate using a commercially available sandwich enzyme-linked immunosorbent assay kit (R&D Systems, Minneapolis, MN).

### Statistical Methods

Qualitative data are expressed as numbers and percentages, and quantitative data as mean, standard deviation (or SEM for sRAGE) or median and interquartile range. To compare baseline characteristics between groups, ANOVA or Kruskal-Wallis test followed respectively by Tukey-Kramer post-hoc test or Dunn’s multiple-comparison test were considered. Proportions were compared among groups using Chi-square test or Fisher’s exact test. Receiver-operating characteristic (ROC) curve was computed and area under the curve was used to evaluate how well the model distinguished SBT success from failure and the presence from the absence of postextubation distress. Confidence intervals (CIs) for areas under receiver-operating characteristic curves were calculated using non-parametric assumptions.

The comparisons of sRAGE between groups at different time-points (before SBT, end SBT and H4 postextubation) were explored in multivariate analysis using mixed model allowing to consider interaction between groups and time-points and random subject effects (random intercept and slope, independent covariance structure). To determine whether plasma levels of sRAGE are associated with lung deaeration based on the LUS score, random-effects model was also considered. Residual normality was checked for all models. All analyses were performed using MedCalc version 10.1.2.0 (Frank Schoonjans, Mariakerke, Belgium) and STATA12 (StataCorp, College Station, TX). A p<0.05 (two-sided) was considered statistically significant.

By design, our study was based on the availability of plasma samples for sRAGE measurements and did not allow a power calculation a priori. Power was calculated a posteriori, with a value of 0.6, favouring type I over type II risk error; therefore our results need to be interpreted cautiously from a clinical perspective.

## Results

### Patients

**Figure 1:**
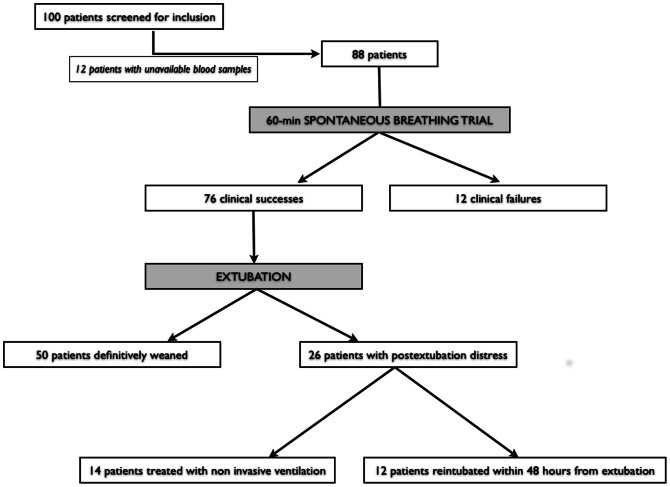
Study flowchart. Patients were screened from a population of weaning patients [5].

Blood samples were available for sRAGE assessment in 88 out of 100 patients enrolled in a previously published study [5], and 88 patients were prospectively included in the present study between february and december 2010 (Figure 1). Baseline patient characteristics are summarized in table 1.

**Table 1:** Baseline Patients Characteristics.

	Overall (N = 88)	SBT failure (n = 12)	SBT success (n = 76)	p	Postextubation success (n = 50)	Postextubation distress(n = 26)	p
**Cause of admission**							
Medical disease, n (%)	40 (45)	3 (25)	37 (49)	0.12	26 (52)	11 (42)	0.42
Surgery, n (%)	40 (45)	5 (42)	35 (46)	0.78	21 (42)	14 (54)	0.33
Multiple trauma, n (%)	8 (9)	4 (33)	4 (5)	0.002	3 (6)	1 (4)	1
**Clinical characteristics**							
Age, years, mean ± SD	60±15	56±17	61±15	0.28	59±15	64±16	0.15
Female gender, n (%)	35 (40)	7 (58)	28 (37)	0.16	19 (38)	9 (35)	0.77
COPD, n (%)	16 (18)	3 (25)	13 (17)	0.51	9 (18)	4 (15)	0.77
Cardiac disease*, n (%)	39 (44)	6 (50)	33 (43)	0.68	19 (38)	14 (54)	0.19
SOFA score at ICU admission, mean ± SD	9.9±3,8	9.8±4	8.8±3,7	0.52	8.6±3.7	9.1±3.9	0.59
SAPS II at ICU admission, mean ± SD	50±16	49±17	50±16	0.88	50±17	48±16	0.66
Creatinine clearance, mL/min, median [IQR]	72 [35–126]	106 [40–114]	70 [35–132]	0.79	76 [38–141]	52 [25–97]	0.24
Prior duration of MV, days, median [IQR]	5 [3–8.5]	6 [4–10.5]	5 [3–7.5]	0.17	4 [2]–[7]	6 [4]–[9]	0.13
Length of total ICU stay, days, median [IQR]	13.5 [7–23.5]	18.5 [12–40]	13 [6.5–21]	0.04	7.5 5–18	19.5 [14]–[27]	<0.001
ICU mortality, n (%)	5 (6)	1 (8)	4 (5)	0.53	1 (2)	3 (12)	0.11
Hospital mortality, n (%)	10 (11)	2 (17)	8 (11)	0.62	2 (4)	6 (23)	0.02
Weight balance since admission (kg), median [IQR]	2 [0–5]	1 [−13–6]	2 [0–5]	0.27	2.3 [0.5–5]	2 [−3–5]	0.44

Data reported as n (%) unless otherwise specified. Percentages may be approximate and their total may not equal 100% due to rounding.

* cardiac disease include coronary heart disease, valvular heart disease, and hypertension.

Abbreviations: SD = standard deviation; IQR = interquartile range; COPD = chronic obstructive pulmonary disease; SOFA = sequential organ failure assessment; SAPS = simplified acute physiologic score; MV = mechanical ventilation; ICU = intensive care unit; LUS = lung ultrasound score.

### Plasma Levels of sRAGE are not Predictive of Successful SBT

Baseline plasma levels of sRAGE were similar between patients who succeeded and those who failed SBT (mean ± SEM, 1021±184 and 1111±714, respectively, p = 0.87) (Table 2). Similar results were found in end-SBT plasma levels of sRAGE between both groups (mean ± SEM, 1038±196 and 1165±92, respectively, p = 0.74) (Table 2).

**Table 2:** Spontaneous breathing trial-induced changes in plasma levels of sRAGE and lung ultrasound score.

	Overall(N = 88)	SBT failure (n = 12)	SBT success (n = 76)	p	Postextubationsuccess (n = 50)	Postextubation distress (n = 26)	p	Overall p
**Plasma level of sRAGE, pg/mL, mean ± SEM**								
*Before SBT*	1099±75	1021±184	1111±82	0.87	1042±86	1245±173	0.38	0.65
*End of SBT*	1148±84	1038±196	1165±92	0.74	1125±92	1239±170	0.61	0.84
*H4 postextubation*	1268±103	–	1268±103	NA	1195±118	1400±196	0.48	0.48
**LUS, mean ± SD**								
*Before SBT*	11.8±5.1	13.1±5.3	11.6±5.1	0.46	9.8±4.6	15±4.1	<0.001	<0.001
*End of SBT*	13.2±5.9	16.4±5.1	12.7±5.9	0.04	10±4.3	18±4.9	<0.001	<0.001
*H4 postextubation*	13.5±6.6	–	13.5±6.6	NA	10.3±5.2	19.6±4.4	<0.001	<0.001

sRAGE = soluble RAGE (receptor for advanced glycation end-products); SD = standard deviation; SEM = standard error of the mean; LUS = lung ultrasound score; SBT = spontaneous breathing trial; NA = non appropriate. All data are presented as mean ± standard error of the mean.

ANOVA or Kruskal-Wallis test followed respectively by Tukey-Kramer post-hoc test or Dunn’s multiple-comparison test were considered for comparisons before SBT (baseline). Multivariate analysis using mixed model (interaction group_x_time as fixed effect, with baseline data included in the model) were considered to measure evolution of sRAGE and LUS (end of SBT and H4 postextubation).

### Plasma Levels of sRAGE are not Predictive of Postextubation Distress after Successful SBT

**Figure 2:**
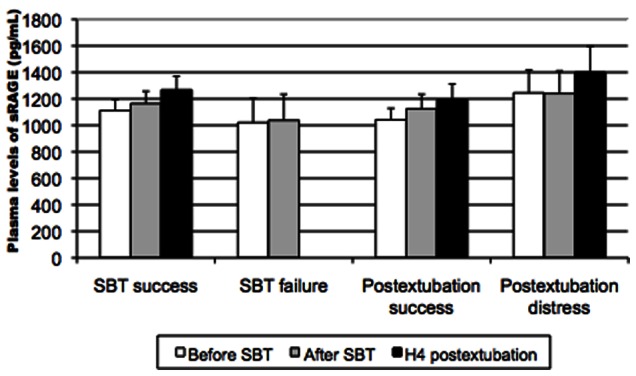
Plasma levels of sRAGE (in pg/mL) measured before, immediately after a spontaneous breathing-trial (SBT) and 4 hours after extubation, and their relations to SBT failure, postextubation distress and successful weaning.

26 patients (34.2% of extubated patients) experienced postextubation distress. Patients with or without postextubation distress had comparable sRAGE levels before SBT (p = 0.38), immediately after SBT (p = 0,64), and 4 hours after extubation (p = 0.48) (Table 2) (Figure 2).

### Plasma Levels of sRAGE are not Associated with Lung Deaeration during Weaning from Mechanical Ventilation

Plasma levels of sRAGE were not associated with lung aeration, as evaluated by LUS score, at baseline, immediately after SBT and 4 hours after extubation in SBT successful patients (p = 0.35, mixed model). As well, changes in plasma levels of sRAGE and those in LUS scores were not correlated, during SBT, during the 4 first hours after SBT, and from inclusion to 4 hours after extubation (p = 0.45, mixed model).

## Discussion

In this prospective observational study of 88 patients weaning from mechanical ventilation, plasma levels of sRAGE, as measured before, immediately after SBT, and 4 hours after extubation in patients who succeeded SBT, were not associated with SBT success or the development of postextubation distress. Moreover, loss in lung aeration, as measured by a lung ultrasound score, was not related to plasma sRAGE, a biomarker for type I alveolar epithelial cell injury. These results, combined with those from a recent study [5], support the hypothesis that weaning failure is associated to excessive lung derecruitment induced by SBT and/or extubation, and that type I alveolar epithelial cell injury is unlikely to account for such a loss of lung aeration.

Obviously, measured plasma sRAGE concentrations in the present study are lower than those from previous studies of sRAGE as a marker of alveolar type I cell injury in patients with ARDS [9], [10]. In patients with ARDS, plasma levels of sRAGE are correlated with clinical and radiographic severity, and decrease over time, suggesting resolution of the injury to the alveolar epithelium [10]. In a large, randomized, controlled trial of lower tidal volume ventilation in ARDS, higher baseline plasma sRAGE levels were associated with worse clinical outcomes in patients randomized to higher tidal volumes, suggesting that patients with high plasma sRAGE were those who benefited most from lung protective ventilation [14]. Our “negative” findings therefore reinforce the hypothesis that plasma sRAGE is associated with the intensity of alveolar damage during ARDS [10], [15], but not in the post-acute care setting, namely during weaning from mechanical ventilation.

The biological rationale for the present study was based on previous findings supporting the hypotheses that sRAGE may be considered a biomarker of lung disease severity [16] and that RAGE may have lung-specific functions distinct from the role of RAGE in other organ systems. One study demonstrated that RAGE enhances the adherence of epithelial cells to collagen-coated surfaces and has a striking capacity for inducing cell spreading, and suggested that RAGE might assist alveolar type I cells to acquire a spreading morphology, thereby ensuring effective gas exchange and alveolar stability [17]. However, putative roles of RAGE in lung homeostasis and/or injury seem unlikely to explain weaning or extubation failure, as revealed by lower plasma sRAGE levels in our study in comparison to those from previous studies in ARDS patients [10], [14]. However, our study did not include the evaluation of alveolar type II epithelial function, which is known to be a key factor to keep lung aeration through the role of surfactant in maintaining alveolar stability (6).

Interestingly, in our study, derecruitment was made of partial loss of lung aeration rather than appearance of new consolidation [5], possibly reinforcing the hypothesis that plasma sRAGE is elevated during diffuse rather than partial loss of aeration. Indeed, lower baseline plasma sRAGE is associated with less diffuse loss of aeration based on computed tomography (CT) lung morphology in ARDS patients [10]. This association of sRAGE with lung aeration was not replicated in weaning patients evaluated with a lung ultrasound score. Reasons for these seemingly contradictory findings may include differences in lung aeration evaluation between lung ultrasound and CT scan [12], [18], a lower level injury to alveolar epithelium in the weaning period as compared to that in the acute period, and/or regulation processes resulting in lower plasma levels of sRAGE in patients under mechanical ventilation for several days [10], [15]. The same reasons may explain, at least in part, the absence of correlation between plasma sRAGE levels and the development of postextubation distress after successful SBT. Postextubation distress has been related to various factors including upper airway obstruction, respiratory failure, alteration of lung resistance or compliance, spontaneous breathing-induced congestive heart failure, aspiration or excessive secretions, encephalopathy or neuromuscular disorders [3].

SBT-induced lung derecruitment may be associated to fluid overload, large pleural effusion, lung superinfection, deterioration of cardiac function, accumulation of abundant bronchial secretions, ventilator-induced diaphragmatic dysfunction [19], [20]. Such derecruitment might also be explained, at least in part, by the development of atelectasis. Thus, unless atelectasis results in diffuse loss of lung aeration [18], [21], sRAGE levels, as well as levels of circulating inflammatory markers, might not be significantly elevated [10], [21], [22].

This study has some limitations. Firstly, plasma sRAGE was not measured in all participants in the original trial, but only in subjects for whom plasma samples were available. Therefore it remains possible that systematic factors contributed in some way to plasma availability. More importantly, our study is not powered enough to avoid a type II error. In order to obtain a power of 80% with a bilateral type I error of 5% in our study, 542 patients would have been needed (n1 = 356; n2 = 186), but differences in sRAGE levels seem relatively low from a clinical perspective and based on available data in such a clinical setting (9, 10, 14, 15, 22), suggesting they may not be clinically significant. Therefore we think our clinical justification is more important in this kind of observational, short time courses, exploratory-type designed, study. Also, the results of this observational study do not account for biological variability, and we unfortunately did not use risk reclassification measures, e.g. by evaluating whether a mixed biomarker and clinical characteristics is a better predictor of postextubation success than the clinical characteristics alone. Unfortunately our results do not include tidal volumes measurements, and for example do not allow the evaluation of rapid shallow breathing index (RSBI).

Secondly, the selection of potential confounders for the analyses was limited to clinical data collected by the original study. Several chronic diseases that may affect clinical outcomes may also impact on sRAGE levels: for example, diabetes mellitus [23] and end stage renal disease [24] have been shown to have elevated levels of plasma RAGE, whereas subjects with coronary artery disease [25], rheumatoid arthritis [26], Alzheimer’s disease [27] and essential hypertension [28] have been shown to have decreased levels of plasma RAGE. Although we were not able to control for these chronic diseases, measured plasma sRAGE levels were consistent with previously reported levels in ICU patients [10]. Thirdly, measurements of sRAGE in the bronchoalveolar lavage (BAL) of our weaning patients were not feasible in our study, and the expression of sRAGE in the fluid of alveolar space remains unknown in this setting. However, previously published data support the hypothesis that the major source of plasma sRAGE is its release from alveolar type I epithelial cell [9], [29]; it seems therefore unlikely that sRAGE levels in the BAL would be different between patients who failed SBT or developed postextubation distress and those who did not in our cohort. Also, the observation time was relatively short, and may not have been sufficient to detect changes in the expression of sRAGE, as measured by ELISA at the protein level. Finally, plasma samples used in this study had been stored for several months at −80°C, as reported in previous studies [9], [14]; whether this extended storage has any effects on plasma biomarker levels remains unknown.

In conclusion, we report the results from the first study aimed at investigating the association of plasma levels of sRAGE, a marker of alveolar type I cell injury, with weaning outcomes in critically ill patients. Based on previously published data, lung aeration loss during SBT or extubation is associated with worse weaning outcomes. This loss seems therefore unlikely to be explained by alveolar epithelial dysfunction, and more work is needed for us to better understand the mechanisms of spontaneous breathing-induced lung derecruitment.

## Supporting Information

Checklist S1 **STROBE Checklist for cohort studies.**
(DOC)

Protocol S1 **Trial Protocol (english and french versions, protocol amendment, ethics committee approval).**
(ZIP)
