# Supplementary material for: Plasma Levels of sRAGE, Loss of Aeration and Weaning Failure in ICU Patients: A Prospective Observational Multicenter Study
Source: PLoS One. 2013 May 27;8(5):e64083. doi: 10.1371/journal.pone.0064083 (PMC3664630; doi:10.1371/journal.pone.0064083)
Supplement: Protocol S1 — Trial Protocol (english and french versions, protocol amendment, ethics committee approval). (ZIP) [file pone.0064083.s002.zip › Study protocol sRAGE_weaning-PLoS/Protocol Pulco ENG.pdf]

Pulmonary and cardiac ultrasound coupled with BNP measurements during weaning from mechanical ventilation

Short Title: PULCO
Version 2: 26/01/2010
No EudraCT/AFSSAPS: 2009-A01310-57
Promoter code: RBHP 2009 PERBET
ClinicalTrials.gov Identifier: NCT01098773

Sponsor
CHU Clermont-Ferrand
58 Rue de Montalembert
63003 Clermont-Ferrand Cedex 1

Principal Investigator:
Dr. Sébastien PERBET
Adult Intensive Care Unit, Anesthesiology & Critical Care Department, Hôtel-Dieu
CHU Clermont-Ferrand
sperbet@chu-clermontferrand.fr
Tel: 04-73-750-501

Methodologist:
Bruno Pereira, PhD (Biostatistics)
Department of Clinical Research and Innovation
CHU Clermont-Ferrand
04 73 754 964

Collaborators:
- Intensive Care Unit (Prof. JM Constantin), Anesthesiology & Critical Care Department (Prof. JE. Bazin), Hôtel-Dieu, CHU Clermont-Ferrand
- Intensive Care Unit (Prof. JJ Rouby), Department of Anesthesiology (Prof. P. Coriat), AP-HP, Groupe Hospitalier Pitie-Salpetriere

SUMMARY

Background:

Mechanical ventilation (MV) is associated with increased morbidity and associated complications increase with the duration of respiratory support. Weaning period is a difficult time and represents 40% of the total duration of mechanical ventilation. The aim is to reduce duration of MV while minimizing the risk of extubation failure. Automated systems seem useful to reduce the duration of weaning but cannot anticipate extubation failure itself. Despite standardization, weaning from MV has a failure rate around 30-35%. Extubation failure, and the need for reventilation during the 48 hours-period following extubation, are clearly associated with increased mortality and morbidity. In a patient being ventilated for more than 48 hours and presenting criteria for extubability, a spontaneous breathing trial (SBT) is conducted in order to approach support-free ventilatory conditions. Arterial blood gases, plasma BNP and echocardiography are proposed to adapt PaO2, monitor PaCO2 or detect cardiac dysfunction. SBT success test allows extubation of the patient.

Our clinical study is designed to evaluate lung ultrasound, echocardiography and plasma BNP levels during the weaning period, in order to assess the relationship between these parameters and SBT or extubation failure. Thus, expected loss of lung aeration during clinical failure could be attributed to cardiac causes. These results could lead to assess prospectively SBT and extubation failures in ICU patients.

Objectives:

Main objective:

To compare the changes in pulmonary aeration during SBT (before, at the end, and 4-6 h after) between patients requiring reventilation before the 48th hour, and those weaned permanently.

Secondary objectives:

- Studying variations, during weaning from mechanical ventilation, of pressure of left ventricle filling (E / Ea) coupled with those of plasma BNP.
- Assessing the effects of E / Ea and BNP variations on the success or failure in the 4-6 early hours.
- Compare the variations of a lung ultrasound score during the test of the weaning from mechanical ventilation between patients failed the spontaneous breathing test (SBT) and those extubated after successful SBT.

Type of study: Biomedical, prospective, observational, open, nonrandomized.

Number of centers:

1. Adult Intensive Care Unit, Prof. Bazin, Hôtel Dieu, CHU Clermont-Ferrand.
2. Intensive care unit, ROUBY Pr, Pr DAR Coriat, AP-HP Pitié-Salpêtrière, Paris

Description of the study:
The eligibility of patients is sought every morning by the ICU medical staff. Patients meeting inclusion criteria are included, after giving their consent or the consent of next of kin.
The measurements are performed in three steps:
- Blood withdrawal before SBT
- At the end of SBT
- After extubation in case of success to SBT

Before and at the end of SBT (1 hour of pressure support ventilation, PSV), we analyze arterial blood gases, BNP plasma levels, and we perform lung and cardiac echography. In case of SBT success, the patient is extubated and arterial blood gases, BNP levels and lung and cardiac ultrasound are evaluated again between H4 and H6.
Patients are followed until the 48th hour after extubation, and patients are then considered as definitily weaned if they need less than 9L/min of oxygen support while breathing spontaneously in order to maintain pulse oximetry above 95%, if they do not need non invasive ventilation (NIV) and if they do not need reintubation.
Primary endpoint:
The primary endpoint is based on the assessment of a pulmonary aeration score *(Lung Ultrasound Score)* before/after SBT, and after extubation.
Secondary endpoints:
The secondary evaluation criteria based on:
- pulmonary aeration score
- left ventricular filling pressure (as determined by cardiac ultrasound)
- plasma BNP levels

Estimated number of patients: 100
Inclusion criteria:
• Age ≥ 18 years
• Duration of mechanical ventilation> 48 hours
• Indwelling arterial catheter
• Patient considered stable under PSV
o pressure support ≤ 8 cmH2O - PEEP ≤ 5 cmH2O - FiO2 ≤ 40% - Spo2 ≥ 95% - Respiratory Rate (RR) ≤ 20/min
- Tidal volume (Vt)> 7 ml/kg of ideal body weight
o Glasgow Coma Scale ≥ 13 and interruption of sedation
o Body temperature < 38°C
o No vasopressor support and a systolic blood pressure (SBP) <160 and >100 mmHg.
• Informed consent from the patient (or from next of kin if the patient is not able to speak)
• Patient under French Social Security system.
Exclusion criteria:
• Refusal of the patient or his designated person of trust
• Tracheotomy
• Trauma with spinal paraplegia above T8 level
• Cardiac arrhythmias (atrial fibrillation) or electrostimulation
• Absence of patient echogenicity at the thoracic level
Benefits and risks of this study:
No risk is expected for enrolled patients. Medical devices used in the study (e.g. ventilators, echography) are widely used in clinical practice. Expected benefits consist in the improvement of knowledge in predicting extubation failure.

LIST OF ABBREVIATIONS

AI: Pressure Support Ventilation
BNP: Brain Natriuretic Peptide
FC: Heart Rate
FiO2: Fraction of Inspired Oxygen
FR: Respiratory
GDS: From Blood Gas
ITV: Full-time speed
OAP: Acute Pulmonary Edema
PaCO2: Blood Pressure CO2
NOT: Systolic Blood Pressure
PEP: Positive Expiratory Pressure
SpO2 Oxygen Saturation
VG: Left Ventricle
NIV: Non-Invasive Ventilation
VS: Spontaneous Ventilation

INDEX

1 Rationale of the study:
1.1 Practice of weaning from mechanical ventilation
1.2 Ultrasound lung and withdrawal
1.3 Cardiac assessment during weaning
1.4 Summary of benefits and risks of research
1.5 Goals and interests of our potential project

2 Objectives
2.1 Main objective
2.2 Secondary objectives

3 Description
3.1 Type of study
3.2 Evaluation Criteria
3.2.1 Primary endpoint
3.2.2 Secondary endpoints

4 Practical realization of the protocol
4.1 Protocol course
4.2 Duration of participation in the study
4.3 Calendar

5 Study population
5.1 Inclusion criteria
5.2 Non-inclusion criteria
5.3 Exclusion criteria
5.4 Exclusion period
5.5 Recruitment procedures

6 Statistical Considerations
6.1 Power calculation
6.2 Statistical treatment of data

7 Management of Adverse Events
7.1 Definitions
7.2 Reporting of serious adverse events
7.3 Monitoring of subjects with an adverse event

8 Patients right of access to documents and data source
8.1 Access to data
8.2 Source data
8.3 Confidentiality

9 Control and quality assurance
9.1 Commitment of the investigators and sponsor
9.2 Insurance
9.3 Control

9.4 CRF

10 Ethical considerations
10.1 Committee to Protect People and authority
10.2 Patient information and consent form written
10.3 Study amendments
10.4 Research support

11 Data processing and storage
11.1 CNIL
11.2 Archives

12 Study budget and insurance
13 Communication - Publishing Rules
14 List of appendices
15 Bibliography

1 Rationale of the study:

Weaning from mechanical ventilation is a critical period in intensive care unit (ICU) patients (1, 2). Weaning failure includes initial spontaneous breathing trial (SBT) failure, postextubation distress and death occuring within 48h following extubation (1). Postextubation distress is defined as reintubation or need for non-invasive ventilation within 48 hours following extubation (1, 3). Following a successful SBT, incidence of reintubation ranges between 3 and 30% (1, 3). Postextubation distress after a successful SBT is associated with increased morbidity and mortality (4). Given the risks associated with delayed or unsuccessful extubation, determining readiness for extubation and predicting postextubation distress is a critical challenge in the ICU. Most of proposed predictors of postextubation distress either require special equipment, or are too complex for bedside use, or have a limited predictive value (3). To date, there are no simple clinical indices known to be powerful predictors of postextubation distress. Many mechanisms may impact on the ability to wean from mechanical ventilation, including spontaneous breathing-induced cardiac failure, and neuromuscular disorders, or alteration of lung resistance and compliance.

1.1 Current Practice of weaning from mechanical ventilation

SBT is performed in patients with all the requirements (SpO2 of 90% or higher under FiO2 less than 40% and positive expiratory pressure (PEEP) of 5 cmH2O or under, hemodynamic stability, Ramsay score of 3 or less, minimal or interrupted sedation, presence of cough, need for less than three tracheal aspirations during the last four hours, no planned procedure requiring sedation or planned surgical intervention.

SBT is run on a T-tube for 1 hour, in order to mimic the conditions under which the patient will be found once extubated. Arterial blood gases, plasma BNP levels and echocardiography may be assessed in order to adapt the level of O2, to monitor PaCO2 monitor or to detect cardiac dysfunction. SBT success (RR <35/min, no chest indrawing, SpO2> 90% with maximal FiO2 of 50% or 9L/min of O2, HR <120 bpm or change in HR<20%, SBP <200mmHg or >80 mmHg, absence of drowsiness or agitation) allows extubation. Following extubation, arterial blood gases, BNP levels and/or echocardiography may help to monitor tolerance.

1.2 Ultrasound lung and withdrawal

The causes of failure of extubation are multiple and sometimes result from complex pathophysiology, including changes in respiratory, cardiac, neuromuscular, metabolic, nutritional, and psychological states. The final alteration in gas exchanges leading to reventilation could be a consequence of a loss in lung aeration. Pulmonary aeration and its variation can be quantified at the bedside by lung ultrasound, a reproducible and non-invasive technique. This technique also helps to assess the type of pulmonary subpleural areas (alveolar or interstitial edema, consolidation, bronchopneumonia images, pleural effusions...). An ultrasound score has been proposed to quantify pulmonary vascular extracellular water (10-16). A significant loss of lung ultrasound aeration could predict SBT failure. In case of SBT success, lung aeration could also predict he need for reventilation.

1.3 Cardiac evaluation during weaning

Cardiogenic pulmonary edema (CPE) is a recognized cause of SBT failure. Mechanical ventilation profoundly alters cardiorespiratory interactions. SBT is associated with the return to a regime of negative intrathoracic pressures that increases venous blood return to the heart. As a result, increased left ventricular preload, as well as afterload, can lead to increased myocardial work. It increases especially because of increased work of breathing and increased sympathetic activity, partly due to anxiety. This phenomenon can lead to CPE-related

hemodynamic dysfunction when left ventricular systolic preexists. However, worsening of diastolic dysfunction alone and variations in left ventricular filling pressures (E/Ea) are not described during SBT. The E / Ea ratio is the ratio of the velocity of diastolic filling of the LV blood pulsed Doppler mitral velocity on displacement of the mitral annulus by pulsed Doppler tissue. BNP (Brain Natriuretic Peptide) is a measurable peptide hormone secreted by cardiac ventricles in response to an increase in ventricular wall stress. This marker is used in clinical practice for the diagnosis of dyspnea resulting from CPE (19). BNP is a reliable marker of left ventricular dysfunction left and its plasma concentrations are correlated to the pressure level of filling (19). BNP monitoring during weaning from MV could be useful to better predict extubation failure (1, 20, 21).

1.4 Summary of the benefits and risks of the research:

No risk is expected for enrolled patients. Medical devices used in the study (e.g. ventilators, echography) are widely used in clinical practice. Expected benefits consist in the improvement of knowledge in predicting extubation failure.

1.5 Goals and potential interests of our project:

Our clinical study is designed to evaluate lung ultrasound, echocardiography and plasma BNP levels during the weaning period, in order to assess the relationship between these parameters and SBT or extubation failure. Thus, expected loss of lung aeration during clinical failure could be attributed to cardiac causes. These results could lead to assess prospectively SBT and extubation failures in ICU patients.

2 Objectives

2.1 Principal objective

To compare the changes in pulmonary aeration during SBT (before, at the end, and 4-6 h after) between patients requiring reventilation before the 48th hour, and those weaned permanently.

2.2 Secondary objectives

- Studying variations, during weaning from mechanical ventilation, of pressure of left ventricle filling (E / Ea) coupled with those of plasma BNP.
- Assessing the effects of E / Ea and BNP variations on the success or failure in the 4-6 early hours.
- Compare the variations of a lung ultrasound score during the test of the weaning from mechanical ventilation between patients failed the spontaneous breathing test (SBT) and those extubated after successful SBT.

3 Description of the study
3.1 Type of study
This is a biomedical research (RBM), prospective, open, non-randomized, two-center, study.

3.2 Evaluation criteria
3.2.1 Primary Outcome
The primary endpoint is the assessment of lung aeration scores Pulmonary *(Lung Ultrasound Score)* test before and after SBT, and after extubation. We will use the lung aeration score proposed and validated by Pr Jean-Jacques Rouby (16). No additional mobilization of the patient is necessary. Each thoracic quadrant is scanned in its entirety by the ultrasound probe, and points are attributed: 0 points = Ventilation normal lung sliding single or "N"; 1 = tails of comets spaced "B1"; 2 = tails comets curtains "B2"; Consolidation 3 = "C". The score is an average score obtained at each phase of weaning. We will also evaluate the variation of aeration in its entirety.

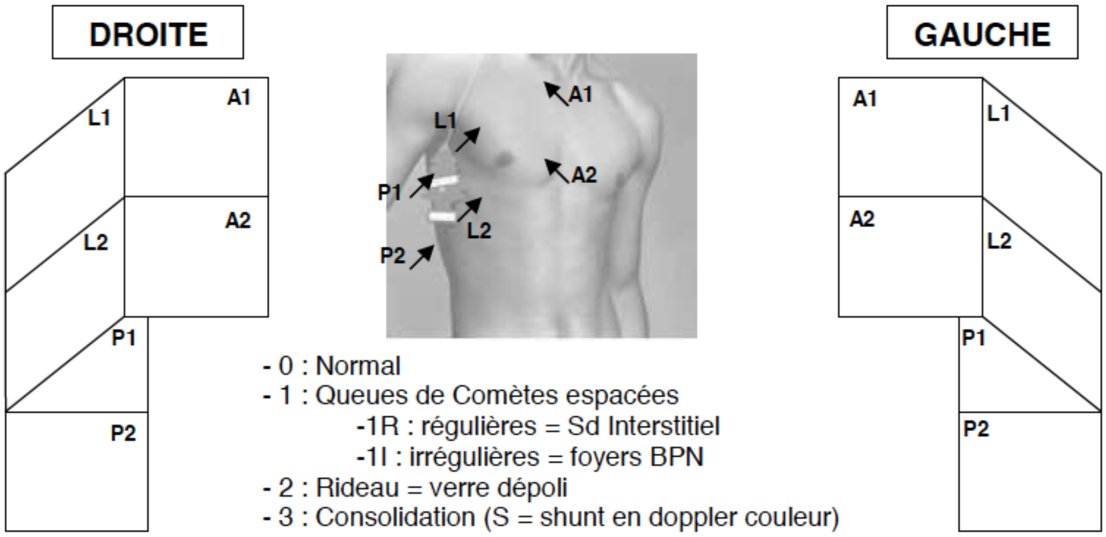

3.2.2 Secondary endpoints:
The secondary evaluation criteria include:
- The lung aeration score, as described in the previous paragraph
- The LV filling pressure (cardiac ultrasound)
- The plasma BNP levels
- Criteria for echocardiography:
  - o Diameter of chamber hunting VG
  - o Kinetics segmental
  - o FRS%
  - o ITV subaortic
  - o mitral pulsed Doppler blood flow: E wave, TDE, A wave
  - o Doppler tissue ring: Ea lateral
  - o ITVmax

4 Practical implementation of the Protocol

This study will be conducted in two centers:

- Intensive Care Unit, Prof. Bazin, Hôtel-Dieu, CHU Clermont-Ferrand
  - Principal Investigator: Dr Sébastien PERBET)
  - Co-Investigators: Prof. Jean-Etienne BAZIN, Dr Jean-Michel Constantin, Dr Sophie Cayot-CONSTANTIN.
- Intensive Care Unit Pr ROUBY, AP-HP, Pitié-Salpêtrière Hospital Group, Paris
  - Principal Investigator: Dr. Alexis SOUMMER
  - Co-Investigators: Prof. Jean-Jacques Rouby, Dr. Qin Lu, Dr. Charlotte Arbelot.

4.1 Procedure protocol

At inclusion, patient demographics, clinical, ventilatory and hemodynamic data are collected (see book collection in the appendix section).

Before SBT, we analyze arterial blood gases, plasma BNP levels, and we perform cardiac and lung echography.

At the end of SBT, we again analyze arterial blood gases, plasma BNP levels and cardiac/lung echography.

Blood sample is gathered from an indwelling arterial catheter, allowing both arterial blood gases analysis and measurements of BNP blood levels.

Echography is a noninvasive technique and is totally harmless to the patient. The use of ultrasound in intensive care is part of routine clinical practice, including weaning period, used to optimize patient management. Echography completion time is around 5 to 10 minutes per patient. The same doctor will perform all ultrasound evaluations at each center: Dr. Sébastien PERBET in Clermont-Ferrand and Dr. Alexis SOUMMER at Pitié-Salpêtrière Hospital.

In case of SBT success, the patient is extubated and analysis of blood gases, BNP levels assessment and cardiac/lung echography are performed at H4-6.

Patients are followed until the 48th hour after extubation, and are considered as finally weaned in the absence of the need for oxygen therapy above 9L/min to maintain SpO2> 95%, in the absence of the need for non-invasive or invasive reventilation (NIV or reintubation).

SBT failure criteria and reventilation criteria:

- RR> 35/min
- Chest indrawing
- Spo2 <90% under FiO2 of 50% or higher, or under O2 therapy flow >9L/min
- HR> 120 bpm or change in HR> 20%
- SBP >200mmHg or <80 mmHg
- Agitation
- Drowsiness

Extubation failure criteria: failure of extubation is an event defined at H48 post-extubation by:

- The need for NIV*:
  - the need for more than 3 hours of NIV per day after abdominal or thoracic surgery
  - in a hypercapnic COPD patient
- O2 mask> 9 l / min for a SpO2> 92%;

- Clinical signs of respiratory distress: RR> 35/min, SpO2 <90% under O2> 9L/min, circulation, exhaustion, poor hemodynamic tolerance, HR> 120, SBP> 160 or <90 mmHg, or poor tolerance with neurological agitation, impaired consciousness;
- The need for reintubation;
- Death within 48 hours after extubation.

** In these situations "prophylactic" NIV is probably advisable (grade 2, consensus conference on NIV; 2006 SRLF-SFAR-SPLF).*

The diagram of the course of study is in Appendix 1.

4.2 Duration of study participation

The total duration of participation in the study for a patient is 48 hours.

4.3 Timetable of the study

- Submission to the ICC Technical Committee of Clermont-Ferrand: 30/11/2009
- Submission to the South East CPP VI (Ethics Committee): January 2010
- Submission to the AFSSAPS: January 2010
- Start of inclusions: favorable opinion from the CCP
- Length of Inclusions: 1 year
- Statistical Report: March 30, 2011
- Final Report: June 31, 2011

5 Population studied

5.1 Inclusion criteria

• Age ≥ 18 years
• Duration of mechanical ventilation> 48 hours
• Indwelling arterial catheter
• Patient considered stable under PSV
o pressure support ≤ 8 cmH2O - PEEP ≤ 5 cmH2O - FiO2 ≤ 40% - Spo2 ≥ 95% - Respiratory Rate (RR) ≤ 20/min, tidal volume (Vt)> 7 ml/kg of ideal body weight
o Glasgow Coma Scale ≥ 13 and interruption of sedation
o Body temperature < 38°C
o No vasopressor support and a systolic blood pressure (SBP) <160 and >100 mmHg.
• Informed consent from the patient (or from next of kin if the patient is not able to speak)
• Patient under French Social Security system

5.2 Non-inclusion criteria

• Refusal of the patient or his designated person of trust
• Tracheotomy
• Trauma with spinal paraplegia above T8 level
• Cardiac arrhythmias (atrial fibrillation) or electrostimulation
• Absence of patient echogenicity at the thoracic level

5.3 Criteria for exclusion

• Respiratory distress due to postextubation laryngeal disease (e.g., airway obstruction, spasm) and requiring reintubation

5.4 Period of exclusion

At the end of patient follow-up in this study, no exclusion period is required.

5.5 Methods of recruitment

The eligibility of patients is sought during the visit and the medical staff who place each morning in the intensive care unit. The final inclusion takes place in the morning for patients meeting the criteria for inclusion and exclusion, with the approval of patient or his designated person of trust.

6 Statistical Considerations

6.1 Power calculation

This study is a pilot study, and data are lacking to establish an expected difference between the two sub-groups on the primary endpoint.
So we justify the feasibility this pilot study by recruitment forecasts and allocation among the sub-groups. Thus, we analyze the data from the observation of 30 patients failing extubation at 48th hour. These patients are in large published series approximately 35% of patients who passed the test of weaning (1-4, 7, 22). SBT failure is found in about 15% of eligible patients in published series (22).
Therefore a total of 100 patients will enable us to study 85 patients passing SBT and thus compare 55 patients without extubation failure with 30 patients failing extubation at 48 hours.

6.2 Processing of statistical data:

Population will be described with regards to demographic and clinical characteristics by percentages or numbers for qualitative and categorical variables, and averages (± standard deviation) or medians (with interquartile ranges) when appropriate.
For primary endpoint analysis, the mean difference before and after SBT will be compared between the patients in the "weaning failure" group and patients in the "successful weaning" group by Student's t test if variables are normally distributed. Normal distribution will be tested by the test Kolmogorov-Smirnov test) and in the case of non-normal distribution, Mann-Whitney (or Wilcoxon) test will be applied. Predictive values of LUS will be determined by ROC (Receiving operating characteristics) curves.
Following tests will applied for secondary endpoints analyses:
- Correlation tests (Pearson / Spearman) for comparisons of changes in LV filling pressure (E/Ea) and BNP levels.
- Comparisons of mean changes in E/Ea and BNP between subgroups (success and failure of weaning).
- Comparisons of mean changes between LUS in patients failing SBT and those in patients passing SBT.

The center-effect will be measured by the coefficient of intra-class correlation and taken into account in the different statistical analyses when necessary.

For all tests, the risk of error is set at α (alpha)=0.05. For each test we will reject the null hypothesis (hypothesis of no difference) for p-values below 0.05. Data will be entered into an Access database, checked, then analyzed using Stata V10 software (Dr Bruno Pereira).

7 Management of adverse events

The investigator has the responsibility to report all adverse events in the observation form.

7.1 Definitions

- Adverse event: an adverse event is defined as any event harmful occurring in a person who participating to biomedical research; this event may be related or unrelated to research or product to which this research applies.
- Adverse reaction: an adverse reaction is any event found undesirable due to research.

We classify serious adverse events in subclasses as follows:

- Expected serious adverse events: when already mentioned in the most recent version of investigator's brochure or in the product characteristics form for authorized drugs, or in the device instructions with CE marking.
- unexpected serious adverse events: if its nature, severity or evolution is inconsistent with information relating to products, performed procedures and methods.
- Serious adverse event: any event or adverse reaction that can result in death or endanger the life of the person participating to the research, in hospitalization or prolongation of hospitalization, in an inability or persistent or significant disability, or in a congenital anomaly or defect.

Death, regardless of cause, including when they correspond to a progression of the disease being treated, are considered as serious events.

Other events that do not meet the qualifications listed above can be considered "potentially serious", including some biological anomalies. The judgment of the investigator or sponsor may lead to report such events in the same way than “serious” event.

7.2 Reporting of serious adverse events

The investigator has to report within 24 hours any event to the promoter

The investigator must determine the causal relationship between the research and serious adverse events. A narrative and informative report must be completed and forwarded to the sponsor. Depending on the nature and severity of the event, anonymous copies of medical records of the patient can be attached, as well as laboratory results.

After receiving the form "serious adverse event", the promoter decides on its accountability over the study and its character unexpected, through a joint analysis with the Regional Pharmacovigilance Centre.

Once a year or on request, the promoter will provide the competent authority and
The Ehics Committee (CPP) with safety report, including all available safety information.
The promoter also send to investigators any information likely to affect the safety of persons.

7.3 Follow-up of subjects presenting an adverse event

Subjects with an adverse event during the study period will be followed as recommended.

8 Right to access to documents and source data

8.1 Access to data

The sponsor is responsible for obtaining the agreement of all parties involved in the research and to ensure direct access to all places of conduct of the research, source data, source documents and reports for the purpose of quality controls or sponsor audits.
The investigators will make available the documents and personal data necessary to study monitoring, quality control and auditing of biomedical research, in accordance with the laws and regulations (Articles L.1121-3 and R.5121-13 of the French Code of Public Health).

8.2 Source data

Source documents are defined as any original document or object that can prove the existence or accuracy of any data or event recorded during the study. These data will be held securely for 15 years by the hospital.

8.3 Data confidentiality

In accordance with the provisions concerning the confidentiality of data that can be accessed (Article L.1121-3 of the French Code of Public Health), people with direct access will take all necessary precautions to ensure the confidentiality of information relating to persons undergoing research, These people, as well as the investigators themselves, are subject to professional secrecy law, under the conditions from Articles 226-13 and 226-14 of the French Criminal Code).
Data will always be transmitted in an anonymous manner. Only the first three letters of the name of the subject and the first two letters of his name will be recorded, along with a coded number indicating specific study inclusion order.
The proponent will ensure that every person included in the research gives written consent for access to personal data

9 Control and Quality Assurance

9.1 Engagements of investigators

The investigator states that this study is conducted in accordance with Good Clinical Practice and Public Health Law n ° 2004-806 (August, 9, 2004). The investigator is also to work in accordance with the Declaration of Helsinki (World Health Organization, Tokyo 2004, revised).

9.2 Quality assurance

A Clinical Research Associate (CRA) mandated by the Proponent will ensure good completion of the study, the collection of data generated by writing their documentation, recording and reporting, in accordance with the Standard Operating Procedures set application within the CHU Clermont-Ferrand and in accordance with Best Practices Clinical as well as laws and regulations.

9.3 Control of quality

The investigator vouches for the authenticity of the data collected under the study and takes legal provisions allowing the sponsor of the study to establish quality control. The Coordinating Investigator and associated investigators therefore agree to make available

during visits of Quality Control performed at regular intervals by the Clinical Research Associate.

9.4 Observation form

All information required by the protocol must be recorded in the observation form and an explanation must be provided for each missing data. The data should be collected as and when they are obtained and transcribed clearly in this form.
Erroneous data recorded on CRFs will be clearly barred. New data will be copied next to the barred information, along with initials, the date and possibly a justification by the investigator or the person authorized to make corrections.

10 Ethical Considerations

10.1 Ethics Committee (*Comite de Protection des Personnes, CPP*)

The protocol information form and consent will be submitted for review to the Ethics Committee (CPP Sud Est VI, CHU Clermont-Ferrand) and to French Health Authority (AFSSAPS). Notification of the favorable opinion from the CPP and the authorization of the AFSSAPS will transmitted to study sponsor.

10.2 Patient Information and Consent Form

Patients should be informed fully and fairly, in understandable terms, of the objectives and constraints of the study, its possible risks and security. They should be reminded of their right to refuse to participate to the study or to withdraw at any time from the study.
All information is present in a patient information form given to the patient. The investigator will collect written informed consent from the patient or from its next-of-kin when appropriate. These documents are approved by the CPP. Two copies will be co-signed both by the investigating physician and the patient. A copy will be given to the patient, the second copy will be collected in the medical record the patient.
If the patient is unable to give consent, the consent of one of its relatives will be sought. Retrospective patient's consent will be searched when possible.

10.3 Amendments to study protocol

Changes to the protocol will be considered substantial or not.
They will by nature be subjected to a new opinion of the Committee for the Protection of People (CPP).

10.4 Research support

Patients care will not differ from current practice. Only cardiac and lung ultrasonography will be performed specifically for the protocol.

11 Data treatment and preservation of documents and data relating to the research

11.1 CNIL

This study is part of the "Reference Method" (MR-001) under provisions of the Act of 6 August 2004 on the protection of individuals with regards to the processing of personal data and amending the Act of 6 January 1978 relating to data, files and freedoms. This change was

approved by decision of 5 January 2006. CHU Clermont-Ferrand, sponsor of the study, signed a commitment to comply with the "Reference Methodology", dated 15/03/2007.

11.2 Archiving

The following documents will be archived in CHU Clermont-Ferrand until study final report:
- Protocol and annexes, amendments thereto,
- Information and consent forms signed originals
- Individual data (authenticated copies of raw data)
- Monitoring documents
- Statistical analyses
- Final study report of the study

At the end of the period of practical utility, all documents to be stored, such as defined in the procedure PG.06.005 "Managing documentation protocols" CHU Clermont-Ferrand will be transferred to the central archives and will be under the responsibility of the Promoter for 15 years after the end of the study, in accordance with institutional practices.
No displacement or destruction can be made without the consent of the Promoter.
At the end of 15 years, the developer will be consulted for destruction. All data, all documents and reports may be subject to audit or inspection.

12 Budget - Insurance

12.1 Budget

The two respective hospital departments endorse costs: print books compendium, realization of ultrasound. Other generated expenses generated do not differ from the usual care the patient. Costs of BNP measurements are supported by the biochemistry laboratory (Pr FIR).

12.2 Insurance

In accordance with regulations, CHU Clermont-Ferrand, as promoter, has purchased liability insurance to guarantee any damages resulting from research at the Hospital Insurance Mutual Company (SHAM), 18 rue Edouard Rochet 69372 Lyon cedex 08. The contract number is 126-016.
It should be noted that non-compliance with legal requirements for research (no opinion from CPP, absence of authorization by AFSSAPS, non consent from the patient…) is an exclusion of warranty.

13 Communication - Publishing Rules

The data will not be disclosed after prior joint agreement of the investigator and sponsor. The results will be communicated. The protocol will be recorded on "Clinical trials.gov."

14 List of appendices

Information and consent form (patient's relatives)
Information and consent form (patient)
Information and retrospective consent form (patient)

15 Bibliography

1. Chien JY, Lin MS, Huang YC, Chien YF, Yu CJ, Yang PC. Changes in B-type natriuretic peptide improve weaning outcome predicted by spontaneous breathing trial. Crit Care Med. 2008 May;36(5):1421-6.
2. Esteban A, Alia I, Tobin MJ, Gil A, Gordo F, Vallverdu I, et al. Effect of spontaneous breathing trial duration on outcome of attempts to discontinue mechanical ventilation. Spanish Lung Failure Collaborative Group. Am J Respir Crit Care Med. 1999 Feb;159(2):512-8.
3. Esteban A, Frutos-Vivar F, Ferguson ND, Arabi Y, Apezteguia C, Gonzalez M, et al. Noninvasive positive-pressure ventilation for respiratory failure after extubation. N Engl J Med. 2004 Jun 10;350(24):2452-60.
4. Antonelli M, Levy M, Andrews PJ, Chastre J, Hudson LD, Manthous C, et al. Hemodynamic monitoring in shock and implications for management. International Consensus Conference, Paris, France, 27-28 April 2006. Intensive Care Med. 2007 Apr;33(4):575-90.
5. Chastre J, Fagon JY. Diagnosis of ventilator-associated pneumonia. N Engl J Med. 2007 Apr 5;356(14):1469; author reply 70-1.
6. Cook DJ, Walter SD, Cook RJ, Griffith LE, Guyatt GH, Leasa D, et al. Incidence of and risk factors for ventilator-associated pneumonia in critically ill patients. Ann Intern Med. 1998 Sep 15;129(6):433-40.
7. Esteban A, Anzueto A, Frutos F, Alia I, Brochard L, Stewart TE, et al. Characteristics and outcomes in adult patients receiving mechanical ventilation: a 28-day international study. Jama. 2002 Jan 16;287(3):345-55.
8. MacIntyre NR, Cook DJ, Ely EW, Jr., Epstein SK, Fink JB, Heffner JE, et al. Evidence-based guidelines for weaning and discontinuing ventilatory support: a collective task force facilitated by the American College of Chest Physicians; the American Association for Respiratory Care; and the American College of Critical Care Medicine. Chest. 2001 Dec;120(6 Suppl):375S-95S.
9. Lellouche F, Mancebo J, Jolliet P, Roeseler J, Schortgen F, Dojat M, et al. A multicenter randomized trial of computer-driven protocolized weaning from mechanical ventilation. Am J Respir Crit Care Med. 2006 Oct 15;174(8):894-900.
10. Frassi F, Gargani L, Tesorio P, Raciti M, Mottola G, Picano E. Prognostic value of extravascular lung water assessed with ultrasound lung comets by chest sonography in patients with dyspnea and/or chest pain. J Card Fail. 2007 Dec;13(10):830-5.
11. Bouhemad B, Zhang M, Lu Q, Rouby JJ. Clinical review: Bedside lung ultrasound in critical care practice. Crit Care. 2007;11(1):205.
12. Agricola E, Picano E, Oppizzi M, Pisani M, Meris A, Fragasso G, et al. Assessment of stress-induced pulmonary interstitial edema by chest ultrasound during exercise echocardiography and its correlation with left ventricular function. J Am Soc Echocardiogr. 2006 Apr;19(4):457-63.
13. Arbelot C, Ferrari F, Bouhemad B, Rouby JJ. Lung ultrasound in acute respiratory distress syndrome and acute lung injury. Curr Opin Crit Care. 2008 Feb;14(1):70-4.
14. Lichtenstein D, Goldstein I, Mourgeon E, Cluzel P, Grenier P, Rouby JJ. Comparative diagnostic performances of auscultation, chest radiography, and lung ultrasonography in acute respiratory distress syndrome. Anesthesiology. 2004 Jan;100(1):9-15.
15. Lichtenstein DA, Meziere GA. Relevance of lung ultrasound in the diagnosis of acute respiratory failure: the BLUE protocol. Chest. 2008 Jul;134(1):117-25.
16. Bouhemad B, Liu ZH, Arbelot C, Zhang M, Ferarri F, Le-Guen M, et al. Ultrasound assessment of antibiotic-induced pulmonary reaeration in ventilator-associated pneumonia*. Crit Care Med. 2009 Jul 23.

17. Lemaire F, Teboul JL, Cinotti L, Giotto G, Abrouk F, Steg G, et al. Acute left ventricular dysfunction during unsuccessful weaning from mechanical ventilation. Anesthesiology. 1988 Aug;69(2):171-9.
18. Wiedemann HP, Wheeler AP, Bernard GR, Thompson BT, Hayden D, deBoisblanc B, et al. Comparison of two fluid-management strategies in acute lung injury. N Engl J Med. 2006 Jun 15;354(24):2564-75.
19. Maisel AS, Krishnaswamy P, Nowak RM, McCord J, Hollander JE, Duc P, et al. Rapid measurement of B-type natriuretic peptide in the emergency diagnosis of heart failure. N Engl J Med. 2002 Jul 18;347(3):161-7.
20. Ait-Oufella H, Tharaux PL, Baudel JL, Vandermeersch S, Meyer P, Tonnellier M, et al. Variation in natriuretic peptides and mitral flow indexes during successful ventilatory weaning: a preliminary study. Intensive Care Med. 2007 Jul;33(7):1183-6.
21. Mekontso-Dessap A, de Prost N, Girou E, Braconnier F, Lemaire F, Brun-Buisson C, et al. B-type natriuretic peptide and weaning from mechanical ventilation. Intensive Care Med. 2006 Oct;32(10):1529-36.
22. Boles JM, Bion J, Herridge M, Marsh B, Mélot C, Pearl R, et al. Weaning from mechanical ventilation. Eur Respir J. 2007 May;29(5):1033-56.
