# Supplementary material for: Plasma Levels of sRAGE, Loss of Aeration and Weaning Failure in ICU Patients: A Prospective Observational Multicenter Study
Source: PLoS One. 2013 May 27;8(5):e64083. doi: 10.1371/journal.pone.0064083 (PMC3664630; doi:10.1371/journal.pone.0064083)
Supplement: Protocol S1 — Trial Protocol (english and french versions, protocol amendment, ethics committee approval). (ZIP) [file pone.0064083.s002.zip › Study protocol sRAGE_weaning-PLoS/Protocole_RBHP_2009_PERBET_Pulco_V2_de_2010_01_26.pdf]

Echographie pulmonaire et cardiaque couplée au BNP lors du sevrage de la ventilation mécanique

Titre abrégé : PULCO

Version : 2 **en date du : 26/01/2010**

Code Promoteur	N° EudraCT ou n° d'enregistrement AFSSAPS
**RBHP 2009 PERBET**	**2009-A01310-57**

Promoteur

C.H.U. de Clermont-Ferrand
58 Rue de Montalembert
63003 Clermont-Ferrand Cedex 1

Investigateur principal :

Monsieur le Docteur PERBET Sébastien
Service de Réanimation Adulte,
Pôle Anesthésie-Réanimation, Hôtel-Dieu
CHU de CLERMONT-FERRAND
sperbet@chu-clermontferrand.fr
Tel : 04-73-750-501

Méthodologiste :

Monsieur Bruno PEREIRA, Docteur en Biostatistique
Délégation Recherche Clinique et Innovation
CHU de CLERMONT-FERRAND
04 73 754 964

Lieux de réalisation de l'étude :

- Service de Réanimation adulte, Pôle Anesthésie-Réanimation, Hôtel-Dieu, CHU de CLERMONT-FERRAND

- Service de Réanimation Polyvalente (Pr J.J. Rouby), Département d'Anesthésie-Réanimation (Pr P. Coriat) AP-HP ; Groupe Hospitalier Pitié-Salpétrière

RESUME

Contexte :

La ventilation mécanique est associée à la survenue de complications dont l'incidence croît avec la durée de l'assistance respiratoire. La période de sevrage ventilatoire reste une période délicate et représente 40% de la durée totale de ventilation mécanique. La procédure de sevrage vise à réduire la durée de la ventilation mécanique tout en minimisant le risque d'échec d'extubation. Des systèmes automatisés paraissent utiles pour réduire la durée de sevrage mais ne permettent pas d'anticiper l'échec d'extubation à proprement parler. Ainsi, malgré la standardisation des procédures de sevrage, 30 à 35 % des patients présentent un échec de sevrage. Cette situation apparaît clairement comme facteur de complication et de surmorbi-mortalité en cas de reventilation dans les 48 heures post-extubation.

Chez un patient ventilé plus de 48 heures et présentant les conditions requises pour une extubation, une épreuve de sevrage est réalisée en ventilation spontanée (VS) sur tube en T pendant 1 heure, permettant de reproduire au mieux les conditions dans lesquelles le patient se trouvera une fois extubé. Les gaz du sang artériels et le BNP plasmatique permettent d'adapter le niveau d'O2, de surveiller la PaCO2 ou de détecter une dysfonction cardiaque associée qu'une échographie cardiaque peut confirmer. La réussite de ce test autorise l'extubation du patient. En post-extubation, les GDS artériels, le BNP et/ou l'échographie cardiaque et pulmonaire permettent de surveiller la bonne tolérance.

Nous proposons donc d'évaluer dans le cadre d'un protocole de recherche biomédicale, en période du sevrage ventilatoire les variations d'aération pulmonaire, d'en préciser l'incidence, et de les quantifier, de les corréler à une éventuelle dysfonction systolique ou diastolique gauche isolée, caractérisée par une augmentation des pressions de remplissage (E/Ea). L'échographie pulmonaire combinée à l'échographie cardiaque, évaluée ici pour la première fois dans cette situation permettra de suivre les variations d'aération pulmonaire pendant l'épreuve de VS/tube. Ainsi, en situation d'échec clinique de sevrage, la perte d'aération pulmonaire attendue à l'échographie pourra être attribuée à une cause cardiologique (et en précisera alors le mécanisme) ou non.

En perspective nous souhaiterions évaluer de manière prospective si ces variations d'aération précoce peuvent prédire l'échec d'extubation à la 48ème heure.

Objectifs :

Objectif principal :

Comparer la variation d'aération pulmonaire au cours du test de sevrage de la ventilation mécanique entre les patients nécessitant une re-ventilation avant la 48ème heure, et ceux sevrés définitivement.

Objectifs secondaires :

Etudier les variations d'aération pulmonaire en fonction des variations de pressions du remplissage VG (E/Ea) couplées à celles du BNP plasmatique.

Evaluer les effets des variations de E/Ea et de BNP sur le succès ou l'échec au cours des 4-6 premières heures.

Comparer la variation d'aération pulmonaire au cours du test de sevrage de la ventilation mécanique entre les patients échouant le test de VS/tube et ceux extubés après réussite du test de VS/tube.

Type d'étude : Recherche biomédicale, prospective, ouverte, non randomisée.

Nombre de centres :

1. Service de Réanimation Adulte, Pr BAZIN, Hôtel Dieu, CHU Clermont-Ferrand.
2. Service de réanimation polyvalente, Pr ROUBY, DAR Pr CORIAT, AP-HP Pitié-Salpétrière, Paris

Description de l'étude :

L'éligibilité des patients est recherchée lors de la visite et du staff médical qui ont lieu chaque matin dans le service de Réanimation. L'inclusion définitive a lieu dans la matinée pour des patients

répondant aux critères d'inclusion et d'exclusion, après consentement du patient ou de sa personne de confiance désignée.

Les mesures sont réalisées à 3 temps :
- Avant le test de sevrage
- En fin de test de sevrage
- Après extubation.

Avant l'épreuve de sevrage en VS avec Aide Inspiratoire (VS-AI et PEEP) et à la fin de l'épreuve de sevrage (VS sur tube en T) (1 heure), nous analysons les gaz du sang artériels, le BNP plasmatique, et réalisons les échographies cardiaque et pulmonaire.

En cas de succès de l'épreuve de sevrage, le patient est extubé et nous renouvelons l'analyse des gaz du sang, du BNP et des échographies cardiaque et pulmonaire à H4-6.

Les patients sont suivis jusqu'à la 48ème heure après l'extubation, et sont considérés comme définitivement sevrés en l'absence de recours à une oxygénothérapie > à 9L/min d'O2 pour maintenir une SpO2> 95%, à la VNI ou à une réintubation.

Critère d'évaluation principal :

Le critère d'évaluation principal repose sur l'appréciation des scores d'aération pulmonaire (*Lung Ultrasound Score*) avant le test de sevrage, en fin de test de sevrage et après extubation.

Critères d'évaluation secondaires :

Les critères d'évaluations secondaires reposent sur :
- le score d'aération pulmonaire, décrit dans le paragraphe précédent
- la pression de remplissage du VG (échographie cardiaque)
- le dosage du BNP plasmatique

Nombre de sujets : 100 patients au total

Critères d'inclusion :
- Age ≥ 18 ans
- Durée de ventilation > 48 HEURES
- Cathéter artériel en place
- Conditions stables en VS AI définies par :
  - AI ≤ 8 - PEP ≤ 5 – FiO2 ≤ 40% - Spo2 ≥ 95% et Fréquence Respiratoire (FR) ≤ 20/min – Volume courant (Vt) > 7 ml/Kg
  - GCS ≥ 13 et arrêt de la sédation
  - T°C < 38 °
  - Pas de catécholamines avec une pression artérielle systolique (PAS)<160 et >100 mmHg.
- Patient ayant donné leur consentement (ou proche du patient si ce dernier n'est pas en mesure de s'exprimer)
- Patient bénéficiant d'un régime de Sécurité Sociale.

Critères de non inclusion :
- Refus du patient ou de sa personne de confiance désignée
- Majeurs protégés et personnes vulnérables
- Patients trachéotomisés
- Traumatismes rachidiens avec paraplégie supérieure à T8
- Troubles du rythme cardiaque (ACFA) ou patients électro-entrainés
- Patient non échogène en transthoracique

Bénéfices et risques de cette étude :

Aucun risque n'est attendu pour les patients participant à cette recherche étant donné qu'il s'agit d'une étude utilisant des appareils médicaux largement utilisés en pratique courante. Le bénéfice attendu concerne l'amélioration des connaissances quant à la prédiction d'échec d'extubation.

LISTE DES ABREVIATIONS

AI : Aide Inspiratoire
BNP : Brain Natriuretic Peptide
FC : Fréquence Cardiaque
FiO2 : Fraction Inspirée d'Oxygène
FR : Fréquence respiratoire
GDS : Gaz Du Sang
ITV : intégrale temps-vitesse
OAP : Œdème Aigu Pulmonaire
PaCO2 : Pression artérielle en CO2
PAS : Pression Artérielle Systolique
PEP : Pression Expiratoire Positive
SpO2 : Saturation en Oxygène
VG : Ventricule Gauche
VNI : Ventilation Non Invasive
VS : Ventilation Spontanée

SOMMAIRE

1 Rationnel de l’étude : 7

1.1 Pratique courante de sevrage de la ventilation mécanique 7

1.2 Echographie pulmonaire et sevrage : 8

1.3 Evaluation cardiaque au cours du sevrage : 8

1.4 Résumé des bénéfices et des risques de la recherche : 9

1.5 Buts et intérêts potentiels de notre projet : 9

2 Objectifs 10

2.1 Objectif principal : 10

2.2 Objectifs secondaires : 10

3 Description de l’étude 10

3.1 Type d’étude : 10

3.2 Critères d’évaluation : 10

3.2.1 Critère d’évaluation principal : 10

3.2.2 Critères d’évaluation secondaires : 11

4 Réalisation pratique du protocole 12

4.1 Déroulement du protocole 12

4.2 Durée de participation à l’étude 14

4.3 Calendrier de l’étude 14

5 Population étudiée : 15

5.1 Critères d’inclusion : 15

5.2 Critères de non inclusion : 15

5.3 Critères d’exclusion : 15

5.4 Période d’exclusion : 16

5.5 Modalités de recrutement : 16

6 Considérations statistiques : 16

6.1 Calcul d’effectif 16

6.2 Traitement statistique des données : 16

7 Gestion des événements indésirables 17

7.1 Définitions 17

7.2 Déclaration des événements indésirables graves 18

7.3 Suivi des sujets ayant présenté un événement indésirable 19

8 Droit d’accès aux données et documents source 19

8.1 Accès aux données 19

8.2 Données source 20

8.3 Confidentialité des données 20

9 Contrôle et assurance de la qualité 21
9.1 Engagement des investigateurs et du promoteur 21
9.2 Assurance de Qualité 21
9.3 Contrôle de Qualité 21
9.4 Cahier d'observation 21
10 Considérations éthiques 22
10.1 Comité de Protection des Personnes et autorité compétente 22
10.2 Information du patient et formulaire de consentement éclairé écrit 22
10.3 Amendements au protocole 23
10.4 Prise en charge relative à la recherche 23
11 Traitement des données et conservation des documents et données relatives à la recherche 23
11.1 CNIL 23
11.2 Archivage 23
12 Budget de l'étude- Assurance 24
13 Communication - Règles de publication 24
14 Bibliographie 25
15 Liste des annexes 27

1 Rationnel de l'étude :

La ventilation mécanique est associée à la survenue de complications dont l'incidence croît avec la durée de l'assistance respiratoire (4-6). La période de sevrage ventilatoire reste une période délicate et représente 40% de la durée totale de ventilation mécanique (7, 8). La procédure de sevrage vise à réduire la durée de la ventilation mécanique tout en minimisant le risque d'échec d'extubation. Des systèmes automatisés paraissent utiles pour réduire la durée de sevrage mais ne permettent pas d'anticiper l'échec d'extubation à proprement parler (1, 2, 9). Ainsi, malgré la standardisation des procédures de sevrage, 30 à 35 % des patients présentent un échec de sevrage et 20 % nécessitent une reventilation dans les 48 heures (1-3). Cette situation apparaît clairement comme facteur de complication et de surmorbi-mortalité (1-3).

1.1 Pratique courante de sevrage de la ventilation mécanique

Un test de sevrage est réalisé chez les patients présentant toutes les conditions requises (SpO2 supérieure ou égale à 90 % avec FiO2 inférieure ou égale à 40 % et PEP inférieure ou égale à 5 cmH2O, stabilité hémodynamique, score de Ramsay inférieur ou égal à 3, sédation arrêtée ou minimale (les traitements antalgiques peuvent être poursuivis), toux audible spontanément ou lors d'une aspiration, nécessité de moins de trois aspirations durant les quatre dernières heures, pas de procédure nécessitant la sédation ni d'intervention chirurgicale programmée) pour une extubation et donc la séparation du respirateur.

Le patient est alors en Ventilation Spontanée avec Aide inspiratoire et une Pression Expiratoire Positive (PEP). Une épreuve de sevrage est réalisée en VS sur tube en T pendant 1 heure, permettant de reproduire au mieux les conditions dans lesquelles le patient se trouvera une fois extubé. Les gaz du sang artériels et le BNP plasmatique permettent d'adapter le niveau d'O2, de surveiller la PaCO2 ou de détecter une dysfonction cardiaque associée qu'une échographie cardiaque peut confirmer. La réussite de ce test (FR<35/min, pas de tirage respiratoire, Spo2 > 90% sous 50% FiO2 ou O2>9L/min, FC < 120 bpm ou variation de FC < 20 %, PAS < 200mmHg ou > 80 mmHg, ni agitation ni somnolence) autorise l'extubation du patient.

En post-extubation, les GDS artériels, le BNP et/ou l'échographie cardiaque et pulmonaire permettent de surveiller la bonne tolérance.

1.2 Echographie pulmonaire et sevrage :

Les causes d'échec d'extubation sont multiples et de physiopathologie parfois complexe incluant les fonctions respiratoires, cardiaques, neuromusculaires centrale et périphérique, métaboliques, nutritionnelles, et enfin l'état psychologique. L'altération finale des échanges gazeux conduisant à la reventilation pourrait donc être une conséquence de perte d'aération pulmonaire. L'aération pulmonaire et sa variation peuvent être quantifiées au lit du patient de manière totalement non invasive et comparée dans le temps, grâce à l'échographie pulmonaire. Cette technique utilisée en réanimation permet en outre d'évaluer le type d'atteinte pulmonaire des zones sous pleurales (oedème interstitiel ou alvéolaire, consolidation, foyers de bronchopneumonie, épanchements pleuraux …). L'analyse du signal échographique a permis d'établir des scores de queues de comètes qui ont été proposés pour quantifier l'eau extra vasculaire pulmonaire (10-16). Une perte importante de cette aération pulmonaire échographie pourrait prédire l'échec de l'épreuve de VS/tube. Inversement en cas de succès de celle-ci, l'étude de la variation d'aération pulmonaire au décours de l'extubation pourrait donc prédire la reventilation du patient.

1.3 Evaluation cardiaque au cours du sevrage :

L'œdème aigu pulmonaire (OAP) cardiogénique est une cause reconnue d'échec de l'épreuve de ventilation spontanée (VS) sur tube. On sait que la ventilation mécanique modifie profondément les interactions cardiorespiratoires. Lors de la mise en VS sur tube, le retour à un régime de pression négative intrathoracique affecte le retour veineux qui augmente. En conséquence, la précharge ventriculaire gauche augmente, de même que sa postcharge étant à l'origine d'une augmentation du travail myocardique. Celui-ci augmente d'autant plus du fait de l'augmentation du travail respiratoire, et de l'augmentation du tonus sympathique en partie liée à l'anxiété. Ce phénomène sera d'autant plus bruyant et conduira à un OAP hémodynamique lorsqu'une dysfonction cardiaque gauche systolique préexiste.

En revanche, l'aggravation d'une dysfonction diastolique isolée ou l'étude de la variation des pressions de remplissage du VG (E/Ea) lors de la mise en VS sur tube ne sont pas décrites alors que des éléments rendent justement compte de l'impact péjoratif d'une balance hydrique positive (17, 18) pouvant favoriser l'apparition d'OAP à fonction systolique conservée lors de cette épreuve. Le rapport E/Ea correspond au rapport de la vélocité du

remplissage protodiastolique du VG en doppler pulsé sanguin mitral sur la vélocité de déplacement de l'anneau mitral en doppler pulsé tissulaire.

Le BNP « Brain Natriuretic Peptide » est une hormone peptidique dosable sécrétée par les ventricules en réponse à une augmentation de stress pariétal de ces mêmes cavités ventriculaires. Ce marqueur est utilisable en pratique clinique pour le diagnostic de dyspnée aigue d'origine cardiaque (19). Le BNP est un marqueur fiable de la dysfonction ventriculaire gauche et leurs concentrations plasmatiques sont corrélées au niveau de pression de remplissage (19). Le monitorage de ces marqueurs au cours du sevrage ventilatoire peut être utile pour mieux prédire l'échec d'extubation (1, 20, 21).

1.4 Résumé des bénéfices et des risques de la recherche :

Aucun risque n'est attendu pour les patients participant à cette recherche étant donné qu'il s'agit d'une étude utilisant des appareils médicaux largement utilisés en pratique courante sans mobilisation supplémentaire du patient.

Le bénéfice attendu concerne l'amélioration des connaissances quant à la prédiction d'échec d'extubation.

1.5 Buts et intérêts potentiels de notre projet :

Nous proposons donc d'évaluer dans le cadre d'un protocole de recherche biomédicale (RBM) en période du sevrage ventilatoire les variations d'aération pulmonaire, d'en préciser l'incidence, et de les quantifier, de les corréler à une éventuelle dysfonction systolique ou diastolique gauche isolée, caractérisée par une augmentation des pressions de remplissage (E/Ea) et une augmentation du BNP.

L'échographie pulmonaire combinée à l'échographie cardiaque, évaluée ici pour la première fois dans cette situation permettra de suivre les variations d'aération pulmonaire pendant l'épreuve de VS/tube. Ainsi, en situation d'échec clinique de sevrage, la perte d'aération pulmonaire attendue à l'échographie pourra être attribuée à une cause cardiologique (et en précisera alors le mécanisme) ou non.

En perspective nous souhaiterions évaluer de manière prospective si ces variations d'aération précoce peuvent prédire l'échec d'extubation à la 48ème heure.

2 Objectifs

2.1 Objectif principal :

Comparer la variation d'aération pulmonaire mesurée avant et après test de sevrage au cours du test de sevrage de la ventilation mécanique entre les patients nécessitant une reventilation avant la 48ème heure, et ceux sevrés définitivement.

2.2 Objectifs secondaires :

- Etudier les variations d'aération pulmonaire en fonction des variations de pressions du remplissage VG (E/Ea) couplées à celles du BNP plasmatique.
- Evaluer les effets des variations de E/Ea et de BNP sur le succès ou l'échec du sevrage au cours des 4-6 premières heures.
- Comparer la variation d'aération pulmonaire mesurée avant et après test de sevrage au cours du test de sevrage de la ventilation mécanique entre les patients échouant le test de VS/tube et ceux extubés après réussite du test de VS/tube.

3 Description de l'étude

3.1 Type d'étude :

Il s'agit d'une étude de recherche biomédicale (RBM) prospective, ouverte, non randomisée, bicentrique.

3.2 Critères d'évaluation :

3.2.1 Critère d'évaluation principal :

Le critère d'évaluation principal repose sur l'appréciation des scores d'aération pulmonaire (*Lung Ultrasound Score*) avant le test de sevrage, en fin de test de sevrage et après extubation, mesurés par échographie pulmonaire.

Nous utilisons le score d'aération proposé et validé dans le service du Pr Jean-Jacques ROUBY (16). Ce score est défini par la somme des points obtenus dans les deux champs

pulmonaire eux-mêmes divisés en 6 quadrants d'étude. Chaque quadrant est balayé dans son intégralité par la sonde d'échographie, et nous attribuons la valeur observée la plus péjorative au quadrant considéré. Aucune mobilisation supplémentaire du patient n'est nécessaire, les coupes postérieures sont obtenues en passant la sonde en latéral juste sous l'aisselle du patient.

0 point = Aération normale, glissement pleural simple ou « N »

1= Queues de comètes espacées « B1 »

2= Queues de comètes en rideaux « B2 »

3= Consolidation « C »

Le score obtenu correspond à une aération moyenne qui sera comparée à chaque phase du sevrage.

Nous évaluons également la variation d'aération dans sa globalité.

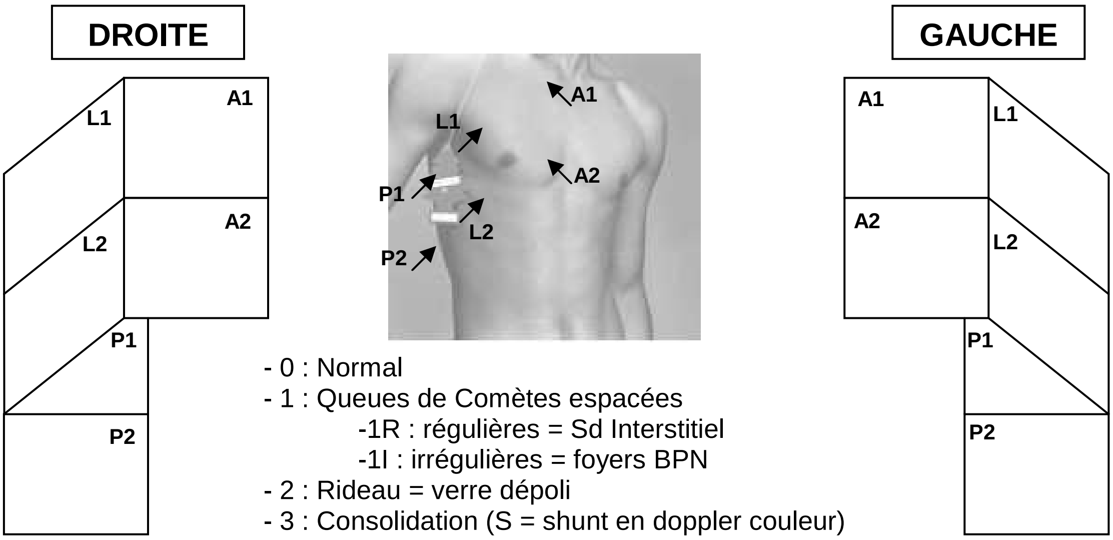

3.2.2 Critères d'évaluation secondaires :

Les critères d'évaluations secondaires seront :

- le score d'aération pulmonaire, décrit dans le paragraphe précédent
- la pression de remplissage du VG (échographie cardiaque)

- le taux plasmatique de BNP
- critères d’échographie cardiaque :
  - Diamètre de la chambre de chasse du VG
  - Cinétique segmentaire
  - FRS %
  - ITV sous aortique
  - Flux mitral doppler pulsé sanguin : onde E, TDE, onde A
  - Doppler tissulaire à l’anneau : Ea latéral
  - Vmax IT

4 Réalisation pratique du protocole

Cette étude se déroulera dans deux centres :

- Service de Réanimation du Pr BAZIN, Hôtel-Dieu, CHU de Clermont-Ferrand
  Investigateur principal : Dr Sébastien PERBET
  Co-investigateurs : Pr Jean-Etienne BAZIN, Dr Jean-Michel CONSTANTIN, Dr Sophie CAYOT-CONSTANTIN.

- Service de Réanimation du Pr ROUBY, AP-HP, Groupe Pitié-Salpétrière, Paris
  Investigateur principal : Dr Alexis SOUMMER
  Co-investigateurs : Pr Jean-Jacques ROUBY, Dr Qin LU, Dr Charlotte ARBELOT.

4.1 Déroulement du protocole

Après l’inclusion du patient, les données démographiques, cliniques, ventilatoires et hémodynamiques sont colligées (voir cahier de recueil en annexe).

- Avant l’épreuve de sevrage en VS avec Aide Inspiratoire (VS-AI et PEEP), nous analysons les gaz du sang artériels, le BNP plasmatique, et réalisons les échographies cardiaque et pulmonaire.

- Epreuve de sevrage en VS sur tube : durée 60 minutes avec ballonnet dégonflé et humidification des voies aériennes assurée par aérosol-thérapie chauffante, débit O2 / pièce en T <9L/min pour maintien Spo2 > 92 %

- A la fin de l’épreuve de sevrage (VS sur tube en T), nous analysons de nouveau les gaz du sang artériels, le BNP plasmatique, et réalisons les échographies cardiaque et pulmonaire.

Les gaz du sang font partie de la pratique usuelle pour guider le clinicien en phase terminale du sevrage (VS/tube) et pour la décision d’extuber le patient et surveiller la bonne tolérance respiratoire en période post-extubation. Ce prélèvement est réalisé sur une ligne de cathéter artériel utilisé pour le monitorage de la pression artérielle.

Cette analyse ne nécessite donc pas de réaliser une ponction vasculaire. Le dosage du BNP se fait sur le même échantillon sanguin et ne nécessite pas de prélèvement supplémentaire.

L’échographie est une technique non invasive et totalement inoffensive pour le patient. Le recours à l’échographie en réanimation fait partie de la pratique courante, y compris en période de sevrage ventilatoire, pour optimiser la prise en charge du patient. Nous réaliserons donc des coupes échographiques pulmonaires et cardiaques transpariétales pour notre étude. Le temps de réalisation est de 5 à 10 minutes. Le même médecin réalisera les 3 échographies successives. Les échographistes réalisant les échographies pulmonaire et cardiaque seront le Docteur Sébastien PERBET à Clermont-Ferrand et le Docteur Alexis SOUMMER à la Pitié-Salpêtrière, tous les 2 titulaires du DIU d’échocardiographie.

En cas de succès de l’épreuve de sevrage, le patient est extubé et nous renouvelons l’analyse des gaz du sang, du BNP et des échographies cardiaque et pulmonaire à H4-6.

Les patients sont suivis jusqu’à la 48ème heure après l’extubation, et sont considérés comme définitivement sevrés en l’absence de recours à une oxygénothérapie > à 9L/min d’O2 pour maintenir une SpO2> 95%, à la VNI ou à une réintubation.

- Critères d’échec de l’épreuve VS/Tube nécessitant la reventilation du patient :

Les signes de mauvaise tolérance sont :

- FR > 35/min
- tirage respiratoire
- Spo2 < 90% sous 50% FiO2 ou O2>9L/min
- FC > 120 bpm ou variation de FC > 20 %
- PAS > 200mmHg ou < 80 mmHg

- agitation
- somnolence.

Un critère suffit à définir un échec.

- Critères d'échec d'extubation : l'échec d'extubation est un évènement défini avant la 48ème heure post-extubation par :
    - la nécessité de recours à la VNI, c'est-à-dire *
        - la nécessité d'introduire la VNI plus de 3H par jour chez un patient de chirurgie abdominale ou thoracique ;
        - ou en contexte médical uniquement chez le patient BPCO hypercapnique ;
    - O2 masque > 9 l/min pour une SpO2 > 92% ;
    - les signes cliniques de détresse respiratoire: FR>35/min, Spo2<90% sous O2 > 9L/min, tirage, épuisement, mauvaise tolérance hémodynamique, FC>120, PAS > 160 ou < 90 mmHg, ou mauvaise tolérance neurologique avec agitation, troubles de conscience ;
    - la nécessité de réintubation ;
    - le décès dans les 48H suivant l'extubation.

** Dans ces situations la VNI est probablement conseillée de manière prophylactique (grade 2, conférence de consensus sur la VNI ; 2006, SRLF-SFAR-SPLF)*

Le schéma du déroulement de l'étude se trouve en annexe 1.

4.2 Durée de participation à l'étude

La durée totale de participation à l'étude pour un patient est de 48 heures. Les seuls examens spécifiques à la recherche sont les échographies cardiaques et pulmonaires, et le dosage systématique du BNP.

4.3 Calendrier de l'étude

- Soumission au Comité Technique du CIC de Clermont-Ferrand : 30/11/2009
- Soumission au CPP Sud Est VI : Janvier 2010
- Soumission à l'AFSSAPS : janvier 2010
- Début des inclusions : dès l'avis favorable du CPP
- Durée des inclusions : 1 an

- Rapport statistique : 30 mars 2011
- Rapport final : 31 juin 2011

5 Population étudiée :

5.1 Critères d'inclusion :

- Age ≥ 18 ans
- Durée de ventilation > 48 HEURES
- Cathéter artériel en place
- Conditions stables en VS AI définies par :
  - AI ≤ 8 - PEP ≤ 5 – FiO2 ≤ 40% - Spo2 ≥ 95% et Fréquence Respiratoire (FR) ≤ 20/min – Volume courant (Vt) > 7 ml/Kg
  - GCS ≥ 13 et arrêt de la sédation
  - T°C < 38 °
  - Pas de catécholamines avec une pression artérielle systolique (PAS) < 160 et > 100 mmHg.
- Patients ayant donné leur consentement selon les modalités décrites par la loi de santé publique du 9 Août 2004, si le patient n'est pas en état d'exprimer sa volonté, c'est une personne de confiance (conjoint, enfant ou autre) qui accepte ou non de signer le formulaire de consentement et donc l'inclusion dans l'étude. Le consentement du patient sera obtenu à posteriori pour l'exploitation des données.
- Patients bénéficiant d'un régime de Sécurité Sociale.

5.2 Critères de non inclusion :

- Refus du patient ou de sa personne de confiance désignée
- Majeurs protégés
- Patients trachéotomisés
- Traumatismes rachidiens avec paraplégie supérieure à T8
- Troubles du rythme cardiaque (ACFA) ou patients électro-entrainés
- Patient non échogène en transthoracique

5.3 Critères d'exclusion :

- Détresse respiratoire laryngée post extubation (obstruction des voies aériennes supérieures) nécessitant la réintubation.

5.4 Période d'exclusion :

A la fin du suivi du patient dans cette étude, aucune période d'exclusion n'est requise.

5.5 Modalités de recrutement :

L'éligibilité des patients est recherchée lors de la visite et du staff médical qui ont lieu chaque matin dans le service de Réanimation. L'inclusion définitive a lieu dans la matinée pour des patients répondant aux critères d'inclusion et d'exclusion, après consentement du patient ou de sa personne de confiance désignée.

6 Considérations statistiques :

6.1 Calcul d'effectif

Cette étude est une étude pilote. Ainsi les données manquent pour établir une différence attendue entre les deux sous-groupes sur le critère principal. Nous justifions donc la faisabilité de cette étude pilote par des prévisions de recrutement et de répartition entres les sous-groupes. Ainsi, nous analyserons les données issues de l'observation de 30 patients en échec d'extubation à la 48ème heure. Ces patients représentent dans les grandes séries publiées environ 35% des patients ayant réussi le test de sevrage (1-4,7,22). Un échec du test de sevrage (épreuve de VS tube) étant retrouvé chez environ 15 % des patients éligibles dans les séries (22), un effectif total de 100 patients doit nous permettre d'étudier 85 patients réussissant le test de sevrage et donc de comparer 55 patients sans échec d'extubation à 30 patients en échec d'extubation à 48 heures.

La centralisation de l'information est réalisée par le Docteur PERBET, investigateur principal, avec mise à jour quotidienne, permettant de décider l'arrêt de l'étude à la 100ème inclusion effective.

6.2 Traitement statistique des données :

La population sera décrite pour toutes ces caractéristiques démographiques et cliniques par des effectifs/pourcentages pour des variables qualitatives et catégorielles et par des moyennes (± écart-type) et des médianes (avec étendu) notamment pour le score d'aération pulmonaire (LUS= *Lung Ultrasound Score*) déterminé pour chaque patient aux 3 temps de l'étude.

Pour l'analyse du critère principal, la différence des moyennes des LUS avant et après test de sevrage seront comparées entre les patients du groupe « échec de sevrage » et les patients du groupe « sevrage réussi » par un test de Student en cas de distribution normale des variables

(distribution gaussienne des données testée par le test de Kolgorov-Smirnov) ; dans le cas de distribution non normale, on utilisera un test de Mann-Whitney (ou Wilcoxon). Une valeur prédictive de LUS sera déterminée par courbe ROC (*receiving operating curve*).

Pour l'analyse des critères secondaires :

- Test de corrélation (Pearson/Spearman) pour comparaison des variations de LUS en fonction des variations de pressions de remplissage VG (E/Ea) couplées à celles du BNP.

- Comparaisons de moyennes de variations de E/Ea et de BNP entre sous-groupes succès et échec de sevrage (tests analogues à ceux de l'analyse principale).

- Comparaisons de moyennes de variations de LUS entre les patients échouant le test de VS/tube et ceux extubés après réussite du test de VS/tube (tests analogues à ceux de l'analyse principale).

Enfin, précisons que l'effet-centre sera mesuré par le coefficient de corrélation intra-classe et pris en compte dans les différentes analyses statistiques si nécessaire.

Pour tous les tests effectués, le risque d'erreur consentie est fixé à α =0,05. Pour chaque test on rejettera l'hypothèse nulle (hypothèse d'absence de différence) pour chaque p-value inférieure à 0,05. Les données seront saisies dans une base ACCESS, vérifiées par un contrôle au dixième et analysées à l'aide du logiciel Stata V10 (par Bruno Pereira).

7 Gestion des événements indésirables

L'investigateur a la responsabilité de rapporter tous les événements indésirables dans le cahier d'observation.

7.1 Définitions

- Evénement indésirable : on définit comme événement indésirable toute manifestation nocive survenant chez une personne qui se prête à une recherche biomédicale, que cette manifestation soit liée ou non à la recherche ou au produit sur lequel porte cette recherche
- Effet indésirable : l'effet indésirable d'une recherche correspond à tout événement indésirable dû à la recherche.

On classe les effets indésirables graves en sous-classes que voici :

- *Effet indésirable grave attendu* : lorsqu'il est déjà mentionné dans la version la plus récente de la brochure pour l'investigateur, ou dans le Résumé des Caractéristiques du Produit

pour les médicaments ayant une Autorisation de Mise sur le Marché, ou dans la notice d'instruction lorsque la recherche porte sur un dispositif médical qui fait l'objet d'un marquage CE.

- *Effet indésirable grave inattendu* : si sa nature, sa sévérité ou son évolution ne concorde pas avec les informations relatives aux produits, actes pratiqués et méthodes utilisées au cours de la recherche.

Evénement ou effet indésirable grave : tout événement ou effet indésirable qui entraîne la mort, met en danger la vie de la personne qui se prête à la recherche, nécessite une hospitalisation ou la prolongation d'une hospitalisation, provoque une incapacité ou un handicap importants ou durables, ou bien se traduit par une anomalie ou une malformation congénitale.

Les décès, quelle que soit leur cause, y compris lorsqu'ils correspondent à une progression de la maladie traitée, sont considérés comme des évènements graves.

D'autres événements ne répondant pas aux qualifications ci-dessus énumérées, peuvent être considérées comme « *potentiellement graves* », notamment certaines anomalies biologiques. Le jugement médical de l'investigateur ou du promoteur pourra conduire à la déclaration de tels événements de la même façon que les événements « graves ».

7.2 Déclaration des événements indésirables graves

L'investigateur a obligation de déclarer dans les 24h au promoteur tout événement indésirable grave survenu chez tout patient inclus dans une étude,

- lors de la phase active de l'étude,
- Après l'arrêt de l'essai, quel que soit le délai, dès lors qu'aucune autre cause que la recherche ne peut raisonnablement être incriminée,

sur un formulaire " Evénement indésirable grave", figurant dans les cahiers d'observation.

L'investigateur doit se prononcer sur la relation de causalité entre l'événement indésirable grave avec la recherche.

Un rapport narratif devra être complété et transmis au promoteur dès l'obtention de nouvelles informations pertinentes. Suivant la nature et la gravité de l'événement, des copies du dossier médical anonymisé du patient peuvent être jointes, ainsi que les résultats des analyses de laboratoire.

A réception du formulaire “ Evénement indésirable grave”, le promoteur analyse cet événement et se prononce sur son imputabilité par rapport à l’étude et sur son caractère inattendu au moyen d’une analyse conjointe avec le Centre Régional de Pharmacovigilance.

Conformément au décret d’application n° 2006-477 du 26/04/2006 modifiant le chapitre I er du titre II du livre 1er de la première partie du code de la santé publique relatif aux recherches biomédicales, toutes les suspicions d’effets indésirables graves inattendus, feront l’objet d’une déclaration du Promoteur à l’autorité compétente, au CPP, dès qu’il en a connaissance et au plus tard 7 jours après la survenue de l’événement.

La survenue et la déclaration des événements indésirables graves seront systématiquement vérifiées lors des visites de monitoring.

Le promoteur tiendra des registres détaillés de tous les événements indésirables qui lui sont notifiés par le ou les investigateurs.

Une fois par an ou sur demande, le promoteur transmettra à l’autorité compétente et au CPP un rapport annuel de sécurité tenant compte de toutes des informations de sécurité disponibles.

Le promoteur transmettra également aux investigateurs de l’étude toute information susceptible d’affecter la sécurité des personnes.

7.3 Suivi des sujets ayant présenté un événement indésirable

Les sujets présentant un événement indésirable durant l’étude seront suivis selon les recommandations de la pratique courante.

8 Droit d’accès aux données et documents source

8.1 Accès aux données

Le promoteur est chargé d’obtenir l’accord de l’ensemble des parties impliquées dans la recherche afin de garantir l’accès direct à tous les lieux de déroulement de la recherche, aux données source, aux documents source et aux rapports dans un but de contrôle de qualité et d’audit par le promoteur.

Les investigateurs mettront à disposition les documents et données individuelles strictement nécessaires au suivi, au contrôle de qualité et à l’audit de la recherche

biomédicale, à la disposition des personnes ayant un accès à ces documents conformément aux dispositions législatives et réglementaires en vigueur (articles L.1121-3 et R.5121-13 du code de la santé publique).

8.2 Données source

Les documents source étant définis comme tout document ou objet original permettant de prouver l'existence ou l'exactitude d'une donnée ou d'un fait enregistrés au cours de l'étude clinique seront conservés pendant 15 ans par l'hôpital.

8.3 Confidentialité des données

Conformément aux dispositions concernant la confidentialité des données auxquelles ont accès les personnes chargées du contrôle de qualité d'une recherche biomédicale (article L.1121-3 du code de la santé publique), les personnes ayant un accès direct prendront toutes les précautions nécessaires en vue d'assurer la confidentialité des informations relatives aux personnes qui se prêtent à la recherche et notamment en ce qui concerne leur identité ainsi qu'aux résultats obtenus.

Ces personnes, au même titre que les investigateurs eux-mêmes, sont soumises au secret professionnel (selon les conditions définies par les articles 226-13 et 226-14 du code pénal).

Pendant la recherche biomédicale ou à son issue, les données recueillies sur les personnes qui s'y prêtent et transmises au promoteur par les investigateurs (ou tous autres intervenants spécialisés) seront rendues anonymes.

Elles ne doivent en aucun cas faire apparaître en clair les noms des personnes concernées ni leur adresse.

Seules les trois premières lettres du nom du sujet et les deux premières lettres de son prénom seront enregistrées, accompagnées d'un numéro codé propre à l'étude indiquant l'ordre d'inclusion des sujets.

Le promoteur s'assurera que chaque personne qui se prête à la recherche a donné son accord par écrit pour l'accès aux données individuelles la concernant et strictement nécessaires au contrôle de qualité de la recherche.

9 Contrôle et assurance de la qualité

9.1 Engagement des investigateurs et du promoteur

L'investigateur s'engage à ce que cette étude soit réalisée en conformité avec les Bonnes Pratiques Cliniques et la loi de santé publique n°2004-806 du 9 août 2004 concernant les recherches biomédicales, le décret d'application n° 2006-477 du 26/04/2006 modifiant le chapitre I er du titre II du livre 1er de la première partie du code de la santé publique relatif aux recherches biomédicales ainsi que les arrêtés en vigueur.

L'investigateur s'engage également à travailler en accord avec la Déclaration d'Helsinki de l'Association Médicale Mondiale (Tokyo 2004, révisée).

9.2 Assurance de Qualité

Un Attaché de Recherche Clinique (ARC) mandaté par le promoteur s'assurera de la bonne réalisation de l'étude, du recueil des données générées par écrit, de leur documentation, enregistrement et rapport, en accord avec les Procédures Opératoires Standards mises en application au sein du CHU de Clermont-Ferrand et conformément aux Bonnes Pratiques Cliniques ainsi qu'aux dispositions législatives et réglementaires en vigueur.

9.3 Contrôle de Qualité

L'investigateur se porte garant de l'authenticité des données recueillies dans le cadre de l'étude et accepte les dispositions légales autorisant le promoteur de l'étude à mettre en place un contrôle de qualité.

- L'investigateur coordinateur et les investigateurs associés acceptent donc de se rendre disponibles lors des visites de Contrôle de Qualité effectuées à intervalles réguliers par l'Attaché de Recherche Clinique.

9.4 Cahier d'observation

Toutes les informations requises par le protocole doivent être consignées sur les cahiers d'observation et une explication doit être apportée pour chaque donnée manquante. Les données devront être recueillies au fur et à mesure qu'elles sont obtenues, et transcrites dans ces cahiers de façon nette et lisible.

Les données erronées relevées sur les cahiers d'observation seront clairement barrées et les nouvelles données seront copiées, à côté de l'information barrée, accompagnées des initiales, de la date et éventuellement d'une justification par l'investigateur ou la personne autorisée qui aura fait la correction.

10 Considérations éthiques

10.1 Comité de Protection des Personnes et autorité compétente

Le protocole, le formulaire d'information et de consentement seront soumis pour avis au Comité de Protection des Personnes Sud Est VI du CHU de Clermont-Ferrand et à l'AFSSAPS.

La notification de l'avis favorable du CPP et l'autorisation de l'AFSSAPS seront transmises au promoteur de l'étude.

10.2 Information du patient et formulaire de consentement éclairé écrit

Les patients seront informés de façon complète et loyale, en des termes compréhensibles, des objectifs et des contraintes de l'étude, des risques éventuels encourus, des mesures de surveillance et de sécurité nécessaires, de leurs droits de refuser de participer à l'étude ou de la possibilité de se rétracter à tout moment. L'investigateur doit également informer les sujets de l'avis rendu par le CPP.

Toutes ces informations figurent sur un formulaire d'information et de consentement remis au patient. Le consentement libre, éclairé et écrit du patient sera recueilli par l'investigateur. Ces documents sont approuvés par le CPP compétent et sont à utiliser pour l'essai concerné, à l'exclusion de tout autre document.

Deux exemplaires originaux seront co-signés par le médecin investigateur et le patient. Un exemplaire sera remis au patient, le second exemplaire conservé dans le dossier médical du patient.

Si le patient n'est pas en mesure de donner son accord, le consentement d'un de ses proches sera recherché. Le consentement rétrospectif du patient sera demandé dans la mesure du possible.

10.3 Amendements au protocole

Les modifications apportées au protocole devront être qualifiées de substantielles ou non. Elles feront, selon leur nature, l'objet d'un nouvel avis du Comité de Protection des Personnes.

10.4 Prise en charge relative à la recherche

La prise en charge des patients ne diffère pas de la pratique courante. Seules une échographie cardiaque et une échographie pulmonaire seront réalisées spécifiquement pour le protocole.

11 Traitement des données et conservation des documents et données relatives à la recherche

11.1 CNIL

Cette étude entre dans le cadre de la « Méthodologie de Référence » (MR-001) en application des dispositions de la loi du 6 août 2004 relative à la protection des personnes physiques à l'égard des traitements de données à caractère personnel et modifiant la loi du 6 janvier 1978 relative à l'informatique, aux fichiers et aux libertés. Ce changement a été homologué par décision du 5 janvier 2006. Le CHU de Clermont-Ferrand, promoteur de l'étude, a signé un engagement de conformité à cette « Méthodologie de Référence » en date du 15/03/2007.

11.2 Archivage

Les documents suivants seront archivés par le nom de l'étude dans les locaux du service de réanimation polyvalent du CHU de Clermont-Ferrand jusqu'à la remise du rapport final de l'étude.

Ces documents sont :

- Protocole et annexes, amendements éventuels,
- Formulaires d'information et consentements originaux signés
- Données individuelles (copies authentifiées de données brutes)
- Documents de suivi
- Analyses statistiques
- Rapport final de l'étude

A l'issue de la période d'utilité pratique, l'ensemble des documents à archiver, tels que définis dans la procédure PG.06.005 « Gestion de la documentation relative aux protocoles » du CHU de Clermont-Ferrand sera transféré aux archives centrales et sera placé sous la

responsabilité du Promoteur pendant 15 ans après la fin de l'étude conformément aux pratiques institutionnelles.

Aucun déplacement ou destruction ne pourra être effectué sans l'accord du Promoteur. Au terme des 15 ans, le promoteur sera consulté pour destruction. Toutes les données, tous les documents et rapports pourront faire l'objet d'audit ou d'inspection.

12 Budget de l'étude - Assurance

12-1 Budget

Les coûts sont pris en charge par les 2 services respectifs : impression des cahiers de recueil, réalisation des échographies (avec l'appareil d'échographie du service et par les médecins du service), Les autres frais générés ne diffèrent pas de la prise en charge habituelle du patient. L'analyse du BNP est prise en charge par le laboratoire de biochimie (Pr SAPIN).

12-2 Assurance

Conformément aux dispositions réglementaires, le CHU de Clermont-Ferrand en sa qualité de promoteur a souscrit une assurance responsabilité civile destinée à garantir les éventuels dommages résultant de la recherche auprès de la Société Hospitalière d'Assurances Mutuelles (SHAM), 18 rue Edouard Rochet 69372 LYON cedex 08. Le numéro de contrat est 126-016.
Il est à noter que le non respect des conditions légales de la recherche (absence d'avis du CPP, absence d'autorisation de l'AFSSAPS, non consentement de la personne, poursuite d'une recherche suspendue ou interdite) est une clause d'exclusion de la garantie.

13 Communication - Règles de publication

Les données ne seront divulguées qu'après accord conjoint préalable de l'investigateur et du promoteur. Les résultats feront l'objet de communications et de publications.

Le protocole sera enregistré sur la base « Clinical trials.gov ».

14 Bibliographie

1. Chien JY, Lin MS, Huang YC, Chien YF, Yu CJ, Yang PC. Changes in B-type natriuretic peptide improve weaning outcome predicted by spontaneous breathing trial. Crit Care Med. 2008 May;36(5):1421-6.
2. Esteban A, Alia I, Tobin MJ, Gil A, Gordo F, Vallverdu I, et al. Effect of spontaneous breathing trial duration on outcome of attempts to discontinue mechanical ventilation. Spanish Lung Failure Collaborative Group. Am J Respir Crit Care Med. 1999 Feb;159(2):512-8.
3. Esteban A, Frutos-Vivar F, Ferguson ND, Arabi Y, Apezteguia C, Gonzalez M, et al. Noninvasive positive-pressure ventilation for respiratory failure after extubation. N Engl J Med. 2004 Jun 10;350(24):2452-60.
4. Antonelli M, Levy M, Andrews PJ, Chastre J, Hudson LD, Manthous C, et al. Hemodynamic monitoring in shock and implications for management. International Consensus Conference, Paris, France, 27-28 April 2006. Intensive Care Med. 2007 Apr;33(4):575-90.
5. Chastre J, Fagon JY. Diagnosis of ventilator-associated pneumonia. N Engl J Med. 2007 Apr 5;356(14):1469; author reply 70-1.
6. Cook DJ, Walter SD, Cook RJ, Griffith LE, Guyatt GH, Leasa D, et al. Incidence of and risk factors for ventilator-associated pneumonia in critically ill patients. Ann Intern Med. 1998 Sep 15;129(6):433-40.
7. Esteban A, Anzueto A, Frutos F, Alia I, Brochard L, Stewart TE, et al. Characteristics and outcomes in adult patients receiving mechanical ventilation: a 28-day international study. Jama. 2002 Jan 16;287(3):345-55.
8. MacIntyre NR, Cook DJ, Ely EW, Jr., Epstein SK, Fink JB, Heffner JE, et al. Evidence-based guidelines for weaning and discontinuing ventilatory support: a collective task force facilitated by the American College of Chest Physicians; the American Association for Respiratory Care; and the American College of Critical Care Medicine. Chest. 2001 Dec;120(6 Suppl):375S-95S.
9. Lellouche F, Mancebo J, Jolliet P, Roeseler J, Schortgen F, Dojat M, et al. A multicenter randomized trial of computer-driven protocolized weaning from mechanical ventilation. Am J Respir Crit Care Med. 2006 Oct 15;174(8):894-900.
10. Frassi F, Gargani L, Tesorio P, Raciti M, Mottola G, Picano E. Prognostic value of extravascular lung water assessed with ultrasound lung comets by chest sonography in patients with dyspnea and/or chest pain. J Card Fail. 2007 Dec;13(10):830-5.
11. Bouhemad B, Zhang M, Lu Q, Rouby JJ. Clinical review: Bedside lung ultrasound in critical care practice. Crit Care. 2007;11(1):205.
12. Agricola E, Picano E, Oppizzi M, Pisani M, Meris A, Fragasso G, et al. Assessment of stress-induced pulmonary interstitial edema by chest ultrasound during exercise echocardiography and its correlation with left ventricular function. J Am Soc Echocardiogr. 2006 Apr;19(4):457-63.
13. Arbelot C, Ferrari F, Bouhemad B, Rouby JJ. Lung ultrasound in acute respiratory distress syndrome and acute lung injury. Curr Opin Crit Care. 2008 Feb;14(1):70-4.
14. Lichtenstein D, Goldstein I, Mourgeon E, Cluzel P, Grenier P, Rouby JJ. Comparative diagnostic performances of auscultation, chest radiography, and lung ultrasonography in acute respiratory distress syndrome. Anesthesiology. 2004 Jan;100(1):9-15.

15. Lichtenstein DA, Meziere GA. Relevance of lung ultrasound in the diagnosis of acute respiratory failure: the BLUE protocol. Chest. 2008 Jul;134(1):117-25.
16. Bouhemad B, Liu ZH, Arbelot C, Zhang M, Ferarri F, Le-Guen M, et al. Ultrasound assessment of antibiotic-induced pulmonary reaeration in ventilator-associated pneumonia*. Crit Care Med. 2009 Jul 23.
17. Lemaire F, Teboul JL, Cinotti L, Giotto G, Abrouk F, Steg G, et al. Acute left ventricular dysfunction during unsuccessful weaning from mechanical ventilation. Anesthesiology. 1988 Aug;69(2):171-9.
18. Wiedemann HP, Wheeler AP, Bernard GR, Thompson BT, Hayden D, deBoisblanc B, et al. Comparison of two fluid-management strategies in acute lung injury. N Engl J Med. 2006 Jun 15;354(24):2564-75.
19. Maisel AS, Krishnaswamy P, Nowak RM, McCord J, Hollander JE, Duc P, et al. Rapid measurement of B-type natriuretic peptide in the emergency diagnosis of heart failure. N Engl J Med. 2002 Jul 18;347(3):161-7.
20. Ait-Oufella H, Tharaux PL, Baudel JL, Vandermeersch S, Meyer P, Tonnellier M, et al. Variation in natriuretic peptides and mitral flow indexes during successful ventilatory weaning: a preliminary study. Intensive Care Med. 2007 Jul;33(7):1183-6.
21. Mekontso-Dessap A, de Prost N, Girou E, Braconnier F, Lemaire F, Brun-Buisson C, et al. B-type natriuretic peptide and weaning from mechanical ventilation. Intensive Care Med. 2006 Oct;32(10):1529-36.
22. Boles JM, Bion J, Herridge M, Marsh B, Mélot C, Pearl R, et al. Weaning from mechanical ventilation. Eur Respir J. 2007 May;29(5):1033-56.

15 Liste des annexes

Annexe 1 : Schéma du déroulement de l’étude
Annexe 2 : Formulaire d’information et de consentement destiné à la famille du patient
Annexe 3 : Formulaire d’information et de consentement des patients
Annexe 4 : Formulaire d’information et de consentement rétrospectif des patients
Annexe 5 : CV des investigateurs
Annexe 6 : Cahier de recueil des données

ANNEXE 1 : Schéma du déroulement de l'étude

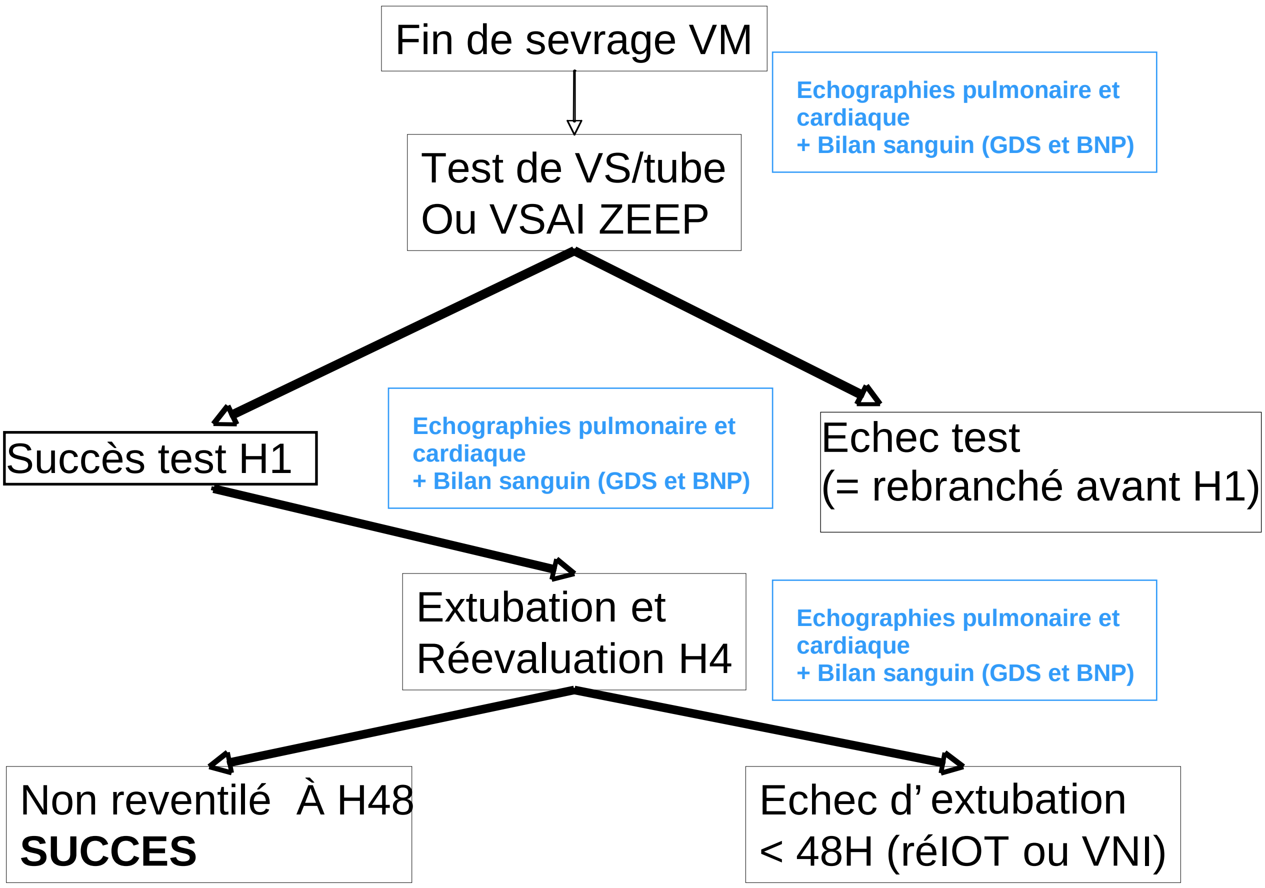

ANNEXE 2 : Formulaire d'information et de consentement destiné à la personne de confiance ou, à un proche, ou à la famille du patient

(à utiliser lorsque le patient n'est pas en mesure de signer à l'entrée dans l'étude)

Echographie pulmonaire et cardiaque couplée au BNP lors du sevrage de la ventilation mécanique

Promoteur :
CHU de Clermont-Ferrand
58 rue Montalembert
63003 Clermont-Ferrand Cedex 1

Investigateur principal :
Dr Sébastien PERBET, Service de réanimation, Hôtel Dieu, CHU Clermont-Fd

Madame, Monsieur,

Vous êtes un membre de la famille (conjoint, ascendant(s), descendant(s), frère(s) ou sœur(s) de plus de 18 ans) d'un patient susceptible de participer à cette étude. Conformément à l'article L 1122-1-2 du Code de la Santé Publique, le consentement du patient peut ne pas être recherché s'agissant d'une recherche qui concerne les patients hors d'état d'exprimer leur consentement. En attendant son rétablissement pour recueillir son consentement, son médecin sollicite votre accord pour débuter la participation du patient à cette étude. Ce document a pour objet de vous éclairer dans le choix que vous ferez pour votre parent. Conformément aux dispositions L 1122-1-2 du Code de la Santé Publique, il recevra l'information nécessaire dès que possible et son consentement sera recherché dès que possible.

Le médecin de la réanimation propose que votre parent participe à une étude concernant la période de sevrage de la ventilation mécanique, c'est-à-dire la période qui permet d'arrêter la respiration artificielle. En effet votre parent a été traité dans le service et a nécessité la mise sous ventilation mécanique. La phase de sevrage du respirateur de la ventilation est une période délicate pour le patient puisque 20 à 35% des patients ne peuvent pas être déconnectés du ventilateur. Ces échecs de sevrage sont parfois lourds de conséquence pour le patient en augmentant la durée de séjour et peuvent aggraver le pronostic. Les causes de ces échecs sont multiples mais un problème cardiaque pourrait avoir une importance déterminante. Pour améliorer la compréhension des mécanismes de ces échecs, et possiblement améliorer la prise en charge des patients concernés, nous souhaitons réaliser cette étude clinique.

Avant d'accepter la participation de votre parent à cette étude, il est important que vous lisiez ce formulaire et que vous en compreniez bien le contenu. Ce formulaire décrit les objectifs, les conditions de réalisation et les contraintes de cette étude. Il décrit également le droit qui est le vôtre et le sien de vous retirer de l'étude à tout moment. Si vous acceptez que votre parent participe, vous recevrez un exemplaire de ce document à conserver.

Cent patients participeront à cette étude, dans le service de Réanimation du Pr BAZIN à l'Hôtel Dieu de Clermont-Ferrand et celui de Réanimation du Professeur ROUBY à la Pitié Salpêtrière, Paris.

Buts de l'étude

L'objectif de cette étude est d'évaluer si le fait de réaliser des examens cardiaques et pulmonaires plus poussés que ce qui est fait habituellement pourrait prédire un éventuel échec de sevrage de la ventilation mécanique.

Durée de participation

La durée de participation à cette étude est de 48 heures.

Déroulement de l'étude

Au cours de cette étude, trois examens seront réalisés systématiquement :

- une échographie pulmonaire
- une échographie cardiaque
- un dosage sanguin du BNP (marqueur de l'insuffisance cardiaque)

Ces examens seront réalisés à trois temps différents :

- avant le début du test de sevrage
- en fin de test de sevrage
- après extubation si le test de sevrage s'avère positif.

L'échographie du poumon et du cœur est aujourd'hui pratiquée en routine dans les services de cardiologique et de Réanimation et est sans danger.

Risques et bénéfices attendus

Aucun risque n'est attendu lors de cette étude étant donné que les examens réalisés font partie de la pratique médicale courante.

Le bénéfice attendu est une amélioration des connaissances concernant le sevrage de la ventilation mécanique et, à terme les résultats permettront d'améliorer la prise en charge du sevrage ventilatoire. Dans notre service, l'évaluation échographique cardiaque et pulmonaire rapprochée fait déjà partie de la prise en charge usuelle des patients.

Participation et interruption de l'étude

La participation à cette étude est entièrement volontaire. Votre refus de faire participer ou votre volonté de faire retirer votre parent de l'étude avant la fin de celle-ci n'aura aucune influence sur la qualité des soins médicaux ultérieurs qui seront prodigués à votre parent.

Sa participation à cette étude pourra être interrompue par son médecin ou par l'investigateur principal de la recherche, sans votre consentement. Si tel était le cas, cela pourrait être dû à l'existence de nouvelles connaissances concernant le sevrage de la ventilation mécanique. Dans ce cas, votre médecin vous en informerait immédiatement.

Réponses à vos questions

Le médecin qui assurera le suivi de votre parent est à votre disposition pour répondre à toutes vos questions sur cette étude ainsi que sur les droits de votre parent.

Confidentialité et traitement informatique

Dans le cadre de cette recherche biomédicale, un traitement informatique des données personnelles de votre proche va être mis en œuvre pour permettre d'analyser les résultats de la

recherche au regard de l'objectif de cette dernière qui vous a été présentée.

A cette fin, les données médicales le concernant, dans la mesure où ces données sont nécessaires à la recherche, seront transmises au Promoteur de la recherche. Ces données seront identifiées par un numéro de code et ses initiales. Ces données pourront également, dans des conditions assurant leur confidentialité, être transmises aux autorités de santé françaises, à d'autres entités du CHU de Clermont Ferrand. Conformément aux dispositions de loi relative à l'informatique aux fichiers et aux libertés, votre proche dispose d'un droit d'accès et de rectification auprès du docteur …………………., investigateur à …………… (Adresse). Vous disposez également d'un droit d'opposition à la transmission des données couvertes par le secret professionnel susceptibles d'être utilisées dans le cadre de cette recherche et d'être traitées.

Votre proche pourra également accéder directement ou par l'intermédiaire d'un médecin de son choix à l'ensemble de ses données médicales en application des dispositions de l'article L. 1111-7 du code de la santé publique. Ces droits s'exercent auprès du médecin qui suit votre proche dans le cadre de la recherche et qui connaît son identité.

Vous êtes libre d'accepter ou de refuser la participation de votre proche à cette recherche. De plus vous pouvez exercer à tout moment votre droit de retrait de cette recherche. Le fait de ne plus participer à cette recherche ne modifiera pas la qualité des soins qui lui sont prodigués. Vous pouvez demander à tout moment des explications complémentaires sur l'étude à l'équipe soignante.

Ce protocole a reçu un avis favorable du Comité de protection des Personnes de la région Sud-Est VI en date du xxx.

En attendant le rétablissement de votre parent et pour autoriser sa participation à cette étude, vous devez vous-même dater et signer le formulaire de consentement joint. Dès que son état le permettra, votre parent sera informé de ce que sa participation à cette étude implique, de la même façon que vous l'avez été. S'il souhaite poursuivre sa participation à cette étude, il devra également dater et signer un formulaire de consentement.

Signature du proche du patient :
(Précédée de la mention « Lu et compris »)

Formulaire de consentement destiné à la personne de confiance ou, à un proche ou, à la famille du patient

ECHOGRAPHIE PULMONAIRE ET CARDIAQUE COUPLEE AU BNP LORS DU SEVRAGE DE LA VENTILATION MECANIQUE

Investigateur principal :
Dr Sébastien PERBET, Service de réanimation, Hôtel Dieu, CHU Clermont-Fd

Je, soussigné(e), (nom, prénom en lettres majuscules)……………………………………….
Né(e) le : ……………………
Demeurant : ……………………………………………………………………………..
déclare :
- que le docteur (nom, prénom, téléphone) ………………….……………………………. a proposé que mon parent ……………………………………………participe à l'étude sus nommée,
- qu'il m'a expliqué en détail le protocole,
- qu'il m'a notamment fait connaître :

- l'objectif, la méthode et la durée de l'étude
- les contraintes et les risques potentiels encourus
- mon droit de refuser de participer et en cas de désaccord de retirer mon consentement à tout moment
- l'obligation d'inscription à un régime de sécurité sociale de mon proche
- que, s'il le souhaite, à son terme, mon proche sera informé(e) par le médecin investigateur de ses résultats globaux
- que le Comité de Protection des Personnes Sud Est VI a émis un avis favorable en date du xxx

Les informations relatives à l'étude recueillies par l'investigateur son traitées confidentiellement. J'accepte :

- que les données enregistrées à l'occasion de cette recherche puissent faire l'objet d'un traitement informatisé anonyme. J'ai bien noté que le droit d'accès prévu par la loi du 6 août 2004 relative à l'informatique, aux fichiers et aux libertés s'exerce à tout moment auprès du médecin qui suit mon proche dans le cadre de la recherche et qui connaît son identité. Je pourrai exercer mon droit de rectification et d'opposition auprès de ce même médecin, qui contactera le promoteur de la recherche.

Mon consentement ne décharge pas les organisateurs de la recherche de leurs responsabilités. Mon parent conserve tous ses droits garantis par la loi.

Après avoir discuté librement et obtenu réponse à toutes mes questions, j'accepte librement et volontairement que mon proche participe à cette recherche biomédicale dans les conditions précisées dans le formulaire d'information et de consentement.

Nom et signature du parent agissant pour le compte ou au nom du patient :
Précédée de la mention « Lu et compris »

Date **(écrite de la main du parent)** :

Lien de parenté :

Nom et signature de l'investigateur :

Date :

Ce document est à réaliser en 2 exemplaires originaux, dont le premier doit être gardé 15 ans par l'investigateur, un autre remis à la personne donnant son consentement

ANNEXE 3 : Formulaire d'information et de consentement du patient

Echographie pulmonaire et cardiaque couplée au BNP lors du sevrage de la ventilation mécanique

Promoteur :
CHU de Clermont-Ferrand
58 rue Montalembert
63003 Clermont-Ferrand Cedex 1

Investigateur principal :
Dr Sébastien PERBET, Service de réanimation, Hôtel Dieu, CHU Clermont-Fd

Madame, Monsieur,

Le médecin de la réanimation vous propose de participer à une étude concernant la période de sevrage de la ventilation mécanique, c'est-à-dire la période qui permet d'arrêter la respiration artificielle. En effet votre traitement dans le service a nécessité la mise en place de la ventilation mécanique. La phase de sevrage de la ventilation est une période délicate pour le patient puisque 20 à 35% des patients ne peuvent pas être déconnectés du ventilateur. Ces échecs de sevrage sont parfois lourds de conséquence pour le patient en augmentant la durée de séjour et peuvent aggraver le pronostic. Les causes de ces échecs sont multiples mais un problème cardiaque pourrait avoir une importance déterminante. Pour améliorer la compréhension des mécanismes de ces échecs, et possiblement améliorer la prise en charge des patients concernés, nous souhaitons réaliser cette étude clinique.

Avant d'accepter votre participation à cette étude, il est important que vous lisiez ce formulaire et que vous en compreniez bien le contenu. Ce formulaire décrit les objectifs, les conditions de réalisation et les contraintes de cette étude. Il décrit également le droit qui est le vôtre de vous retirer de l'étude à tout moment. Si vous acceptez de participer à cette étude, vous recevrez un exemplaire de ce document à conserver.

Cent patients participeront à cette étude, dans le service de Réanimation du Pr BAZIN à l'Hôtel Dieu, Clermont-Ferrand et celui de Réanimation du Professeur ROUBY à la Pitié Salpêtrière, Paris.

Buts de l'étude

L'objectif de cette étude est d'évaluer si le fait de réaliser des examens cardiaques et pulmonaires plus poussés que ce qui est fait habituellement pourrait prédire un éventuel échec de sevrage de la ventilation mécanique.

Durée de participation

La durée de participation à cette étude est de 48 heures.

Déroulement de l'étude

Au cours de cette étude, trois examens seront réalisés systématiquement :

- une échographie pulmonaire

- une échographie cardiaque
- un dosage sanguin du BNP (marqueur de l'insuffisance cardiaque)

Ces examens seront réalisés à trois temps différents :

- avant le début du test de sevrage
- en fin de test de sevrage
- après extubation si le test de sevrage s'avère positif.

Risques et bénéfices attendus

Aucun risque n'est attendu lors de cette étude étant donné que les examens réalisés font partie de la pratique courante.

Le bénéfice attendu est une amélioration des connaissances concernant le sevrage de la ventilation mécanique et, à terme les résultats permettront d'améliorer la prise en charge du sevrage ventilatoire. Dans notre service, l'évaluation échographique cardiaque et pulmonaire rapprochée fait déjà partie de la prise en charge usuelle des patients.

Participation et interruption de l'étude

La participation à cette étude est entièrement volontaire. Votre refus de participer ou votre volonté de retirer votre consentement avant la fin de l'étude n'aura aucune influence sur la qualité des soins médicaux ultérieurs qui vous seront prodigués.

Votre participation à cette étude pourra être interrompue par votre médecin ou par l'investigateur principal de la recherche, sans votre consentement. Si tel était le cas, cela pourrait être dû à l'existence de nouvelles connaissances concernant le sevrage de la ventilation mécanique. Dans ce cas, votre médecin vous en informerait immédiatement.

Réponses à vos questions

Le médecin qui assurera votre suivi est à votre disposition pour répondre à toutes vos questions sur cette étude.

Confidentialité et traitement informatique

Dans le cadre de cette recherche biomédicale, un traitement informatique des données personnelles vous concernant va être mis en œuvre pour permettre d'analyser les résultats de la recherche au regard de l'objectif de cette dernière qui vous a été présenté.

A cette fin, vos données médicales, dans la mesure où ces données sont nécessaires à la recherche, seront transmises au Promoteur de la recherche. Ces données seront identifiées par un numéro de code et vos initiales. Ces données pourront également, dans des conditions assurant leur confidentialité, être transmises aux autorités de santé françaises, à d'autres entités du CHU de Clermont Ferrand. Conformément aux dispositions de loi relative à l'informatique aux fichiers et aux libertés, vous disposez d'un droit d'accès et de rectification auprès du docteur …………………., investigateur à …………… (Adresse). Vous disposez également d'un droit d'opposition à la transmission des données couvertes par le secret professionnel susceptibles d'être utilisées dans le cadre de cette recherche et d'être traitées.

Vous pouvez également accéder directement ou par l'intermédiaire d'un médecin de votre choix à l'ensemble de vos données médicales en application des dispositions de l'article L. 1111-7 du code de la santé publique. Ces droits s'exercent auprès du médecin qui vous suit dans le cadre de la recherche et qui connaît votre identité.

Vous êtes libre d'accepter ou de refuser la participation à cette recherche. De plus vous pouvez exercer à tout moment votre droit de retrait de cette recherche. Le fait de ne plus participer à cette recherche ne modifiera pas la qualité des soins qui vous sont prodigués.

Vous pouvez demander à tout moment des explications complémentaires sur l'étude à l'équipe soignante.

Ce protocole a reçu un avis favorable du Comité de protection des Personnes de la région Sud-Est VI en date du xxx.

Lorsque vous aurez lu cette note d'information et obtenu les réponses aux questions que vous vous posez en interrogeant le médecin investigateur, il vous sera proposé, si vous en êtes d'accord, de donner votre consentement écrit en signant le document préparé à cet effet.

Signature du patient :
(Précédée de la mention « Lu et compris »)

Formulaire de consentement du patient

ECHOGRAPHIE PULMONAIRE ET CARDIAQUE COUPLEE AU BNP LORS DU SEVRAGE DE LA VENTILATION MECANIQUE

Investigateur principal :
Dr Sébastien PERBET, Service de réanimation, Hôtel Dieu, CHU Clermont-Fd

Je, soussigné(e), (nom, prénom en lettres majuscules)………………………………………….
Né(e) le : ……………………
Demeurant : ……………………………………………………………………………………..
déclare :
- que le docteur (nom, prénom, téléphone) …………………..…………………………………….. m'a proposé de participer à l'étude sus nommée,
- qu'il m'a expliqué en détail le protocole,
- qu'il m'a notamment fait connaître :

- l'objectif, la méthode et la durée de l'étude
- les contraintes et les risques potentiels encourus
- mon droit de refuser de participer et en cas de désaccord de retirer mon consentement à tout moment
- mon obligation d'inscription à un régime de sécurité sociale
- que, si je le souhaite, à son terme, je serais informé(e) par le médecin investigateur de ses résultats globaux
- que le Comité de Protection des Personnes Sud Est VI a émis un avis favorable en date du xxx

Les informations relatives à l'étude recueillies par l'investigateur son traitées confidentiellement. J'accepte :

- que les données enregistrées à l'occasion de cette recherche puissent faire l'objet d'un traitement informatisé anonyme. J'ai bien noté que le droit d'accès prévu par la loi du 6 août 2004 relative à l'informatique, aux fichiers et aux libertés s'exerce à tout moment auprès du médecin qui suit mon proche dans le cadre de la recherche et qui connaît son identité. Je pourrai exercer mon droit de rectification et d'opposition auprès de ce même médecin, qui contactera le promoteur de la recherche.

Mon consentement ne décharge pas les organisateurs de la recherche de leurs responsabilités. Je conserve tous mes droits garantis par la loi.

Après avoir discuté librement et obtenu réponse à toutes mes questions, j'accepte librement et volontairement de participer à cette recherche biomédicale dans les conditions précisées dans le formulaire d'information et de consentement.

Nom et signature du patient :
Précédée de la mention « Lu et compris »

Date **(écrite de la main du patient)** :

Nom et signature de l'investigateur :

Date :

Ce document est à réaliser en 2 exemplaires originaux, dont le premier doit être gardé 15 ans par l'investigateur, un autre remis à la personne donnant son consentement

ANNEXE 4 : Formulaire d'information et de consentement rétrospectif du patient

Echographie pulmonaire et cardiaque couplée au BNP lors du sevrage de la ventilation mécanique

Promoteur :
CHU de Clermont-Ferrand
58 rue Montalembert
63003 Clermont-Ferrand Cedex 1

Investigateur principal :
Dr Sébastien PERBET, Service de réanimation, Hôtel Dieu, CHU Clermont-Fd

Madame, Monsieur,

Le médecin de la réanimation vous propose de participer à une étude concernant la période de sevrage de la ventilation mécanique, c'est-à-dire la période qui permet d'arrêter la respiration artificielle. En effet votre traitement dans le service a nécessité la mise en place de la ventilation mécanique. La phase de sevrage de la ventilation est une période délicate pour le patient puisque 20 à 35% des patients ne peuvent pas être déconnectés du ventilateur. Ces échecs de sevrage sont parfois lourds de conséquence pour le patient en augmentant la durée de séjour et peuvent aggraver le pronostic. Les causes de ces échecs sont multiples mais un problème cardiaque pourrait avoir une importance déterminante. Pour améliorer la compréhension des mécanismes de ces échecs, et possiblement améliorer la prise en charge des patients concernés, nous souhaitons réaliser cette étude clinique.

Avant d'accepter de poursuivre votre participation à cette étude, il est important que vous lisiez ce formulaire et que vous en compreniez bien le contenu. Ce formulaire décrit les objectifs, les conditions de réalisation et les contraintes de cette étude. Il décrit également le droit qui est le vôtre de vous retirer de l'étude à tout moment. Si vous acceptez de participer à cette étude, vous recevrez un exemplaire de ce document à conserver.

Cent patients participeront à cette étude, dans le service de Réanimation du Pr BAZIN à l'Hôtel Dieu, Clermont-Ferrand et celui de Réanimation du Professeur ROUBY à la Pitié Salpêtrière, Paris.

Buts de l'étude

L'objectif de cette étude est d'évaluer si le fait de réaliser des examens cardiaques et pulmonaires plus poussés que ce qui est fait habituellement pourrait prédire un éventuel échec de sevrage de la ventilation mécanique.

Durée de participation

La durée de participation à cette étude est de 48 heures.

Déroulement de l'étude

Au cours de cette étude, trois examens seront réalisés systématiquement :

- une échographie pulmonaire

- une échographie cardiaque
- un dosage sanguin du BNP (marqueur de l'insuffisance cardiaque)

Ces examens seront réalisés à trois temps différents :
- avant le début du test de sevrage
- en fin de test de sevrage
- après extubation si le test de sevrage s'avère positif.

Risques et bénéfices attendus

Aucun risque n'est attendu lors de cette étude étant donné que les examens réalisés font partie de la pratique courante.

Le bénéfice attendu est une amélioration des connaissances concernant le sevrage de la ventilation mécanique et, à terme les résultats permettront d'améliorer la prise en charge du sevrage ventilatoire. Dans notre service, l'évaluation échographique cardiaque et pulmonaire rapprochée fait déjà partie de la prise en charge usuelle des patients.

Participation et interruption de l'étude

La participation à cette étude est entièrement volontaire. Votre volonté de retirer votre consentement avant la fin de l'étude n'aura aucune influence sur la qualité des soins médicaux ultérieurs qui vous seront prodigués.

Votre participation à cette étude pourra être interrompue par votre médecin ou par l'investigateur principal de la recherche, sans votre consentement. Si tel était le cas, cela pourrait être dû à l'existence de nouvelles connaissances concernant le sevrage de la ventilation mécanique. Dans ce cas, votre médecin vous en informerait immédiatement.

Réponses à vos questions

Le médecin qui assurera votre suivi est à votre disposition pour répondre à toutes vos questions sur cette étude.

Confidentialité et traitement informatique

Dans le cadre de cette recherche biomédicale, un traitement informatique des données personnelles vous concernant va être mis en œuvre pour permettre d'analyser les résultats de la recherche au regard de l'objectif de cette dernière qui vous a été présenté.

A cette fin, vos données médicales, dans la mesure où ces données sont nécessaires à la recherche, seront transmises au Promoteur de la recherche. Ces données seront identifiées par un numéro de code et vos initiales. Ces données pourront également, dans des conditions assurant leur confidentialité, être transmises aux autorités de santé françaises, à d'autres entités du CHU de Clermont Ferrand. Conformément aux dispositions de loi relative à l'informatique aux fichiers et aux libertés, vous disposez d'un droit d'accès et de rectification auprès du docteur …………………., investigateur à …………… (Adresse). Vous disposez également d'un droit d'opposition à la transmission des données couvertes par le secret professionnel susceptibles d'être utilisées dans le cadre de cette recherche et d'être traitées.

Vous pouvez également accéder directement ou par l'intermédiaire d'un médecin de votre choix à l'ensemble de vos données médicales en application des dispositions de l'article L. 1111-7 du code de la santé publique. Ces droits s'exercent auprès du médecin qui vous suit dans le cadre de la recherche et qui connaît votre identité.

Vous êtes libre d'accepter ou de refuser la poursuite de la participation à cette recherche. De plus vous pouvez exercer à tout moment votre droit de retrait de cette recherche. Le fait de ne plus participer à cette recherche ne modifiera pas la qualité des soins

qui vous sont prodigués. Vous pouvez demander à tout moment des explications complémentaires sur l'étude à l'équipe soignante.

Ce protocole a reçu un avis favorable du Comité de protection des Personnes de la région Sud-Est VI en date du xxx.

Lorsque vous aurez lu cette note d'information et obtenu les réponses aux questions que vous vous posez en interrogeant le médecin investigateur, il vous sera proposé, si vous en êtes d'accord, de donner votre consentement écrit en signant le document préparé à cet effet.

Signature du patient :
(Précédée de la mention « Lu et compris »)

Formulaire de consentement rétrospectif du patient

ECHOGRAPHIE PULMONAIRE ET CARDIAQUE COUPLEE AU BNP LORS DU SEVRAGE DE LA VENTILATION MECANIQUE

Investigateur principal :
Dr Sébastien PERBET, Service de réanimation, Hôtel Dieu, CHU Clermont-Fd

Lors de votre admission dans le service, en raison de la gravité de votre état de santé et de l'urgence médicale, nous avons été dans l'impossibilité de vous demander votre consentement par écrit pour la participation à ce protocole de recherche clinique.

Conformément à la loi (art. L. 1122-1 du Code de la Santé Publique), vous avez été quand même inclus dans l'étude le …../…../…../. Quand cela a été possible, c'est à une personne (parent, proche) qui vous accompagnait lors de votre arrivée au CHU, qu'a été demandé l'accord de votre participation à cette recherche, ce qu'il (elle) a accepté.

Je, soussigné(e), (nom, prénom en lettres majuscules)………………………………………….
Né(e) le : ……………………
Demeurant : ……………………………………………………………………………………..
déclare :
- que le docteur (nom, prénom, téléphone) …………………..…………………………………….. m'a proposé de participer à l'étude sus nommée,
- qu'il m'a expliqué en détail le protocole,
- qu'il m'a notamment fait connaître :
  - l'objectif, la méthode et la durée de l'étude
  - les contraintes et les risques potentiels encourus
  - mon droit de refuser de participer et en cas de désaccord de retirer mon consentement à tout moment
  - mon obligation d'inscription à un régime de sécurité sociale
  - que, si je le souhaite, à son terme, je serais informé(e) par le médecin investigateur de ses résultats globaux
  - que le Comité de Protection des Personnes Sud Est VI a émis un avis favorable en date du xxx

Les informations relatives à l'étude recueillies par l'investigateur son traitées confidentiellement. J'accepte :

- que les données enregistrées à l'occasion de cette recherche puissent faire l'objet d'un traitement informatisé anonyme. J'ai bien noté que le droit d'accès prévu par la loi du 6 août 2004 relative à l'informatique, aux fichiers et aux libertés s'exerce à tout moment auprès du médecin qui suit mon proche dans le cadre de la recherche et qui connaît son identité. Je pourrai exercer mon droit de rectification et d'opposition auprès de ce même médecin, qui contactera le promoteur de la recherche.

Mon consentement ne décharge pas les organisateurs de la recherche de leurs responsabilités. Je conserve tous mes droits garantis par la loi.

Après avoir discuté librement et obtenu réponse à toutes mes questions, j'accepte librement et volontairement de poursuivre la participation à cette recherche biomédicale dans les conditions précisées dans le formulaire d'information et de consentement.

Nom et signature du patient :
Précédée de la mention « Lu et compris »

Date **(écrite de la main du patient)** :

Nom et signature de l'investigateur :

Date :

Ce document est à réaliser en 2 exemplaires originaux, dont le premier doit être gardé 15 ans par l'investigateur, un autre remis à la personne donnant son consentement

ANNEXE 5 : CV des investigateurs

NOM : …**PERBET**………….. PRENOM(S) : **Sébastien**………………..

ADRESSE PROFESSIONNELLE : Service de Réanimation Adulte, Pôle Anesthésie et Réanimation Hôtel-Dieu, CHU Clermont-Ferrand, Bd L. Malfreyt, 63058 Clermont-Ferrand, France

TELEPHONE(S) : I_0 I_4_I I_7_I_3_I I_7_I_5_I I_0_I_5_I I_0_I_1_I

FAX : I_0 I_4_I I_7_I_3_I I_7_I_5_I I_0_I_5_I I_0_I_0_I

Adresse courriel : sperbet@chu-clermontferrand.fr

DATE DE NAISSANCE : I_1_I_4_I I_0_I_7_I I_1_I_9_I_7_I_7_I

ANNEE d'obtention du DOCTORAT EN MEDECINE : I_2_I_0_I_0_I_7_I

N° d'inscription au CONSEIL DE L'ORDRE : **63/5575**

N° ADELI: **63 10 5575 3**

SPECIALITE : Anesthésie-Réanimation

FONCTIONS ACTUELLES :

- HOPITAL - Assistant des hopitaux

……………………………………………………………………

PARTICIPATION ANTERIEURE A DES RECHERCHES BIOMEDICALES (en particulier dans le domaine de la recherche visée, publications éventuelles) :

-Etudes multicentriques :

-Cathedia

- ATRYN : Etude phase II/III sur AT3 et sepsis

Publications

[1] F. Lagneau, **S. Perbet**, D. Delefosse, A. Wernet, J. Stocco, J. Marty. Drugs phamacokinetics in ICU patients : consequences of hypoalbuminemia upon drugs monitoring and dosing scheme. ***Intensive Care medicine***,2004 Jun;30(6):1247.

[2] **S. Perbet**, P. Trouiller, C. Paugam-Burtz, M.-H. Denninger, J. Mantz. Nadroparin-induced skin necrosis then thrombocytosis in intensive care unit: difficulty in diagnosis. Nécrose cutanée puis thrombocytose induite par la nadroparine : un diagnostic inhabituel. ***Ann Fr Anesth Reanim***. **2007**;26(9):791-4.

[3] **S. Perbet**, F. Lagneau, A. Wernet, D. Delefosse, J. Belghiti, J. Marty . Incidence des demandes d'examens biologiques hors prescription médicale écrite en réanimation post-opératoire de chirurgie digestive lourde. ***Ann Fr Anesth Reanim***. **2007**.

[4] F. Wallet, **S. Perbet**, M.-H. Fléron, V. De Castro, G. Godet, M. Bertrand, E. Kieffer, J.-P. Goarin, P. Coriat. Elephant trunk prosthesis kinking: Transesophageal echocardiography diagnosis. **Anesthesia Analgesia 2008.**

[5] **Sébastien Perbet**, Florent Wallet, Victor De Castro, Olivier Ducombs, Akthar Rama, Jean-Pierre Goarin, Pierre Coriat. Early echocardiographic diagnosis of aorto-right ventricular fistula during emergent surgery for penetrating precordial trauma. ***J Trauma 2009***.

DATE : I_1_I_6_I I_1_I_1_I I_2_I_0_I_0_I_9_I SIGNATURE : ***S PERBET***

NOM : …**SOUMMER** PRENOM(S) : **Alexis**………………..
ADRESSE PROFESSIONNELLE : Service de Réanimation Polyvalente Adulte, CHU Pitié-Salpétrière, 47-83 Bd de l'hopital, 75013 Paris, France
TELEPHONE(S) : 01 42 17 73 17

FAX : 01 42 17 73 01

Adresse courriel : alexis.soummer@psl.aphp.fr

DATE DE NAISSANCE : 21 / 08 / 1978

ANNEE d'obtention du DOCTORAT EN MEDECINE : I_2_I_0_I_0_I_8_I

N° d'inscription au CONSEIL DE L'ORDRE : **75 75032**

N° ADELI: **75 17 5032 4**

SPECIALITE : Anesthésie-Réanimation

FONCTIONS ACTUELLES :

- HOPITAL Chef de Clinique Assistant des hôpitaux

………………………………………………………………….

PARTICIPATION ANTERIEURE A DES RECHERCHES BIOMEDICALES
(en particulier dans le domaine de la recherche visée, publications éventuelles) :

-Etudes multicentriques :

-Cathedia

<u>Publications</u>

1. "Emergency treatment of tracheobronchial stent migration", Anesthesiology 2004; Nov
2. "Splenic artery aneuvrism", Rev Prat 2006; Feb
3."Failure o fdeferasirox, an rion chelator agent combined with antifungals in severe zygomycosis" , Antimicrob Agents Chemother 2008, April

DATE : I_1_I_6_I I_1_I_1_I I_2_I_0_I_0_I_9_I SIGNATURE : ***A SOUMMER***

NOM : ROUBY… PRENOM(S) : Jean-Jacques

ADRESSE PROFESSIONNELLE : Service de Réanimation Polyvalente Adulte, CHU Pitié-Salpétrière, 47-83 Bd de l'hopital, 75013 Paris, France

TELEPHONE(S) : 0142177300

FAX : 01 42 17 73 01

jean-jacques.rouby@psl.aphp.fr

DATE DE NAISSANCE : 17 09 46

ANNEE d'obtention du DOCTORAT EN MEDECINE 1973

N° d'inscription au CONSEIL DE L'ORDRE : 75/29406

N° ADELI:

SPECIALITE : Anesthésie-Réanimation

FONCTIONS ACTUELLES : Chef de Service de la Réanimation Polyvalente du Département d'Anesthésie-Réanimation (Pr Coriat)

- HOPITAL Pitié-Salpêtrière 75013 Paris

………………………………………………………………

PARTICIPATION ANTERIEURE A DES RECHERCHES BIOMEDICALES

(en particulier dans le domaine de la recherche visée, publications éventuelles) :

Publications (2 dernières années)

- B. Bouhemad, F. Ferrari, K. Leleu, C. Arbelot, Q. Lu, **J.J. Rouby**. Echocardiographic Doppler estimation of pulmonary artery pressure in critically ill patients. *ANESTHESIOLOGY* 108: 55-62, **2008**
- **J.J. Rouby**. Variable Effect of Positive End-expiratory Pressure on Oxygenation in Patients with Acute Respiratory Distress Syndrome. *ANESTHESIOLOGY*. 108:165-6, **2008**.
- C. Arbelot, F. Ferrari, B. Bouhemad and **J.J. Rouby**. Lung ultrasound in acute respiratory distress syndrome and acute lung injury. *CURRENT OPINION IN CRITICAL CARE* 14: 70–74, **2008**
- B. Bouhemad, A. Nicolas-Robin, C. Arbelot, M. Arthaud,; F. Féger, **J-J Rouby**. Isolated and reversible impairment of ventricular relaxation in patients with septic shock. *CRITICAL CARE MEDICINE*.36: 766-774, **2008**
- **J. J. Rouby**, F. Ferrari, B. Bouhemad and Q. Lu. The quest for optimal positive end-expiratory pressure continues. *CRITICAL CARE* **12**:408 (doi:10.1186/cc6208) , **2008**
- F. Ferrari, Z.H. Liu, Q. Lu, M.H. Becquemin, K. Louchahi, G. Aymard, C.H. Marquette, **J.J. Rouby**. Comparison of lung tissue concentrations of nebulized ceftazidime in ventilated piglets: ultrasonic versus vibrating plate nebulizers. *INTENSIVE CARE MEDICINE* 34: 1718-1723 **2008**
- B. Bouhemad, A. Nicolas-Robin, C. Arbelot, M. Arthaud, F. Féger; **J.J. Rouby**. Acute Left Ventricular Dilatation and Shock-Induced Myocardial Dysfunction. *CRITICAL CARE MEDICINE*.37 (in press), **2009**
- L. Brochard and **J.J. Rouby**. Changing Mortality in Acute Respiratory Distress Syndrome? Yes, We Can! *AMERICAN JOURNAL OF RESPIRATORY AND CRITICAL CARE MEDICINE* 179:177-178, **2009**
- B. Bouhemad , Z.H. Liu, C. Arbelot , M. Zhang, F. Ferarri , M. Le-Guen, M. Girard, Q. Lu, **J.J. Rouby**. .*CRITICAL CARE MEDICINE*. Jul 23. [Epub ahead of print] **2009**
- F. Ferrari, C. Girardi, O. Petitjean, C.H. Marquette, F. Wallet, **J.J. Rouby** and the Experimental ICU Study Group. Nebulized ceftazidime in experimental pneumonia caused by partially resistant Pseudomonas aeruginosa. *INTENSIVE CARE MEDICINE*. 35: 1792-1800, **2009**
- J. Kesecioglu, R. Beale, T. Stewart, G.P. Findlay, **J.J. Rouby**, L. Holzapfel, P. Bruins, E.J. Steenken, O.K. Jeppesen, B. Lachmann. Exogenous Natural Surfactant for Treatment of Acute Lung Injury and the Acute Respiratory Distress Syndrome. *AMERICAN JOURNAL OF RESPIRATORY AND CRITICAL CARE MEDICINE* 180: 989-994, **2009**

DATE : 20 11 2009 SIGNATURE :

Jean-Jacques ROUBY
[illegible] Polyvalente - Chef de Service
[illegible] Réanimation Pr CORIAT
GHUE Groupe Hospitalier Pitié-Salpêtrière Pole 3
47-83, Bd de l'Hôpital - 75013 PARIS
Tél. : 01 42 17 73 00 - Fax : 01 42 17 73 26

NOM : …LU PRENOM(S) : Qin

ADRESSE PROFESSIONNELLE : Service de Réanimation Polyvalente Adulte, CHU Pitié-Salpétrière, 47-83 Bd de l'hopital, 75013 Paris, France
TELEPHONE(S) : 0142178438

FAX : 01 42 17 73 01

Adresse courriel qin.lu@psl.aphp.fr

DATE DE NAISSANCE : le 12 décembre 1963

ANNEE d'obtention du DOCTORAT EN MEDECINE 1987

N° d'inscription au CONSEIL DE L'ORDRE : 75/65647

N° ADELI:

SPECIALITE : Anesthésie-Réanimation

FONCTIONS ACTUELLES : Praticien hospitalier

- HOPITAL :

… La Pitié-Salpêtrtière, 75013, Paris
…………………………………………………………………

PARTICIPATION ANTERIEURE A DES RECHERCHES BIOMEDICALES
(en particulier dans le domaine de la recherche visée, publications éventuelles) :

Publications (2 dernières années)

1. **Lasocki S,** Lu Q, **Sartorius A, Fouillat D, Remerand F, Rouby JJ Open and Closed-circuit Endotracheal Suctioning in Acute Lung Injury: Efficiency and Effects on Gas Exchange.** *Anesthesiology* **2006, 104 (1):39-47**
2 Lu Q**, Constantin JM, Nieszkowska A, Elman M, Vieira S and Rouby JJ: Measurement of alveolar derecruitment in patients with acute lung injury: computerized tomography versus pressure-volume curve.** *Crit Care* **2006; 10: R95**.
3 **Sartorius A,** Lu Q, **Vieira S, Tonnellier M, Lenaour G, Goldstein I and Rouby JJ: Mechanical ventilation and lung infection in the genesis of air-space enlargement.** *Crit Care* **2007; 11: R14**.
4 **Remerand F, Luce V, Badachi Y,** Lu Q**, Bouhemad B and Rouby JJ:Incidence of chest tube malposition in the critically ill: a prospective computed tomography study.** *Anesthesiology* **2007 Jun;106(6):1112-9.**
5 Bouhemad B, Zhang M, **Lu Q**, Rouby JJ. Bedside lung ultrasound in critical care practice. ***Crit Care*** 2007; 11(1): 205**.**

6 Rouby JJ, Ferrari F, Bouhemad B , **Lu Q.** Positive end-expiratory pressure in acute respiratory distress syndrome: should the 'open lung strategy' be replaced by a 'protective lung strategy'? ***Crit Care*** 2007; 11: 180.

7 Bouhemad B, Ferrari F, Leleu K, Arbelot C, **Lu Q,** Rouby JJ. Echocardiographic Doppler estimation of pulmonary artery pressure in critically ill patients with severe hypoxemia. ***Anesthesiology*** 2008; 108: 55-62.

8 Ferrari F Liu ZH, Lu Q, **Becquemin MH, Louchahi K, Aymard G, Marquette CH, Rouby JJ : Comparison of lung tissue concentrations of nebulized ceftazidime in ventilated piglets: ultrasonic versus vibrating plate nebulizers.** *Intensive Care Med* ***2008*****; 34:17:18-23**.

9 Bouhemad B, Liu ZH, Arbelot C, Zhang M, Ferarri F, Leguen M, Lu Q, **Rouby JJ: Lung ultrasound assessment of antibiotic-induced pulmonary re-aeration in ventilator–associated pneumonia.** *Crit Care Med***, 35: 1792-8002009**

10 Ferrari F, **Lu Q**, Girardi C, Tonnelier M, Goldstein I, Petit-Jean O, Wallet O;, Marquette CH and Rouby JJ. Nebulized ceftazidime in experimental pneumonia caused by partially resistant *Pseudomonas aeruginosa*. ***Intensive Care Med***, 2009

DATE : le 20 novembre 2009 SIGNATURE :

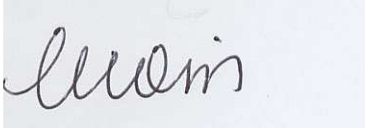

NOM ARBELOT PRENOM(S) : Charlotte Maria Bianca

ADRESSE PROFESSIONNELLE : Service de Réanimation Polyvalente Adulte, CHU Pitié-Salpétrière, 47-83 Bd de l'hopital, 75013 Paris, France

TELEPHONE(S) : Portable 0673534382

FAX : 01 42 17 73 01

Adresse courriel charlotte.arbelot@psl.aphp.fr

DATE DE NAISSANCE : 15/10/1976

ANNEE d'obtention du DOCTORAT EN MEDECINE 2005

N° d'inscription au CONSEIL DE L'ORDRE : 75/72156

N° ADELI: 751721564

SPECIALITE : Anesthésie-Réanimation
FONCTIONS ACTUELLES :Praticien Hospitalier temps plein

- HOPITAL CHU Pitié-Salpétrière

……………………………………………………………………

PARTICIPATION ANTERIEURE A DES RECHERCHES BIOMEDICALES
(en particulier dans le domaine de la recherche visée, publications éventuelles) :

Participation à plusieurs études internationales et multicentriques de phase II et de phase III.

<u>Publications (2 dernières années)</u>

1.Ultrasound assessment of antibiotic-induced pulmonary reaeration in ventilator-associated pneumonia*

Bouhemad B, Liu ZH, Arbelot C, Zhang M, Ferarri F, Le-Guen M, Girard M, Lu Q, Rouby JJ.

Crit Care Med. 2009 Jul 23. [Epub ahead of print]

2.Acute left ventricular dilatation and shock-induced myocardial dysfunction.

Bouhemad B, Nicolas-Robin A, Arbelot C, Arthaud M, Féger F, Rouby JJ.

Crit Care Med. 2009 Feb;37(2):441-7.

3.Isolated and reversible impairment of ventricular relaxation in patients with septic shock.

Bouhemad B, Nicolas-Robin A, Arbelot C, Arthaud M, Féger F, Rouby JJ.

Crit Care Med. 2008 Mar;36(3):766-74.

4.Lung ultrasound in acute respiratory distress syndrome and acute lung injury.

Arbelot C, Ferrari F, Bouhemad B, Rouby JJ.

Curr Opin Crit Care. 2008 Feb;14(1):70-4. Review.

5.Echocardiographic Doppler estimation of pulmonary artery pressure in critically ill patients with severe hypoxemia.

Bouhemad B, Ferrari F, Leleu K, Arbelot C, Lu Q, Rouby JJ.

Anesthesiology. 2008 Jan;108(1):55-62.

DATE : 20/11/2009 SIGNATURE :

CURRICULUM VITAE – INVESTIGATEUR PRINCIPAL

ETAT CIVIL
NOM : BAZIN
PRENOM : JEAN-ETIENNE

ADRESSE PROFESSIONNELLE
HOPITAL : CHU Clermont-Ferrand
SERVICE :Anesthésie-Réanimation
ADRESSE :Hôtel-Dieu, Boulevard Léon Malfreyt
CODE POSTAL :63058 VILLE : Clermont-Ferrand
PAYS : FRANCE
TELEPHONE : 0473750475 FAX :04 73 75 04 74

TITRES : Professeur des Universités – Praticiens Hospitalier.

FONCTIONS PROFESSIONNELLES
Mention de la modalité d'exercice (libéral/salarié) en cas d'exercice mixte. Préciser dans quel cadre se déroulera la fonction d'investigateur :
Chef de service d'anesthésie-Réanimation, salarié exclusif.
En cas d'exercice hospitalier, préciser si l'investigateur est le chef de service, à défaut, il est nécessaire de joindre une autorisation appropriée signée du chef de service, autorisant l'investigateur à réaliser la recherche dans son service :

NUMERO D'INSCRIPTION A LORDRE DES MEDECINS
NUMERO :6303308
CONSEIL DEPARTEMENTAL DE L'ORDRE DES MEDECINS DE : Puy de Dômes

QUALIFICATION RECONUE PAR L'ORDRE DES MEDECINS,
AVEC ANNEE D'OBTENTION : Anesthésie-Réanimation 1987

PUBLICATIONS PERTINENTES AU REGARD DE LA RECHERCHE ENVISAGEE

Futier E, Constantin JM, Combaret L, Mosoni L, Roszyk L, Sapin V, Attaix D, Jung B, Jaber S, Bazin JE. Pressure support ventilation attenuates ventilator-induced protein modifications in the diaphragm. Crit Care. 2008;12:R116.
Constantin JM, Jaber S, Futier E, Cayot-Constantin S, Verny-Pic M, Jung B, Bailly A, Guerin R, Bazin JE. Respiratory effects of different recruitment maneuvers in acute respiratory distress syndrome. Crit Care. 2008;12:R50.
Constantin JM, Cayot-Constantin S, Roszyk L, Futier E, Sapin V, Dastugue B, Bazin JE, Rouby JJ. Response to recruitment maneuver influences net alveolar fluid clearance in acute respiratory distress syndrome. Anesthesiology. 2007;106:944-51.
Jabaudon M, Bonnin M, Bolandard F, Chanseaume S, Dauphin C, Bazin JE. Takotsubo syndrome during induction of general anaesthesia. Anaesthesia. 2007;62:519-23.
Constantin JM, Schneider E, Cayot-Constantin S, Guerin R, Bannier F, Futier E, Bazin JE. Remifentanil-based sedation to treat noninvasive ventilation failure: a preliminary study. Intensive Care Med. 2007;33 :82-7.
Bonnin M, Therre P, Albuisson E, Beaujard H, Barthelemy I, Mondie JM, Bazin JE. Comparison of a propofol target-controlled infusion and inhalational sevoflurane for fibreoptic intubation under spontaneous ventilation. Acta Anaesthesiol Scand. 2007; 51:54-9.

DATE 26 Novembre 2009 SIGNATURE

NOM : …**CONSTANTIN**………….. PRENOM(S) : **Jean-Michel**………………..

ADRESSE PROFESSIONNELLE : Service de Réanimation Adulte, Pôle Anesthésie et Réanimation Hôtel-Dieu, CHU Clermont-Ferrand, Bd L. Malfreyt, 63058 Clermont-Ferrand, France

TELEPHONE(S) : I_0 I_4_I I_7_I_3_I I_7_I_5_I I_0_I_5_I I_0_I_1_I

FAX : I_0 I_4_I I_7_I_3_I I_7_I_5_I I_0_I_5_I I_0_I_0_I

Adresse courriel : jmconstantin@chu-clermontferrand.fr

DATE DE NAISSANCE : I_2_I_2_I I_0_I_1_I I_1_I_9_I_7_I_1_I

ANNEE d'obtention du DOCTORAT EN MEDECINE : I_1_I_9_I_9_I_8_I

N° d'inscription au CONSEIL DE L'ORDRE : **63/4662**

N° ADELI: **631046620**

SPECIALITE : Anesthésie-Réanimation

FONCTIONS ACTUELLES :

- HOPITAL	- Praticien Hospitalier, responsable UF réanimation (PH)
- UNIVERSITE	Ph.D.
	…………………………………………………………………

5 Publications internationales

1. Futier E, Constantin JM, Combaret L, et al. Pressure support ventilation attenuates ventilator-induced protein modifications in the diaphragm. Critical care (London, England) 2008;12:R116.
2. Constantin JM, Roszyk L, Guerin R, et al. [Tolerance of caspofungine in intensive care unit: a prospective study]. Ann Fr Anesth Reanim 2008;27:819-24.
3. Constantin JM, Jaber S, Futier E, et al. Respiratory effects of different recruitment maneuvers in acute respiratory distress syndrome. Critical care (London, England) 2008;12:R50.
4. Constantin JM, Schneider E, Cayot-Constantin S, et al. Remifentanil-based sedation to treat noninvasive ventilation failure: a preliminary study. Intensive care medicine 2007;33:82-7.
5. Constantin JM, Cayot-Constantin S, Roszyk L, et al. The Response to Recruitment Maneuver Influences Net Alveolar Fluid Clearance in Acute Respiratory Distress Syndrome. Anesthesiology 2007;106:944-51.

DATE : I_2_I_6_I I_0_I_5_I I_2_I_0_I_0_I_9_I SIGNATURE :***JM Constantin***

CV résumé d'investigateur

NOM : **CAYOT-CONSTANTIN**………….. PRENOM(S) : **Sophie**………………..

ADRESSE PROFESSIONNELLE : Service de Réanimation Adulte, Pôle Anesthésie et Réanimation Hôtel-Dieu, CHU Clermont-Ferrand, Bd L. Malfreyt, 63058 Clermont-Ferrand, France

TELEPHONE(S) : I_0 I_4_I I_7_I_3_I I_7_I_5_I I_0_I_5_I I_0_I_1_I

FAX : I_0 I_4_I I_7_I_3_I I_7_I_5_I I_0_I_5_I I_0_I_0_I

Adresse courriel : scayot@chu-clermontferrand.fr

DATE DE NAISSANCE : I_2_I_2_I I_1_I_2_I I_1_I_9_I_7_I_3_I

ANNEE d'obtention du DOCTORAT EN MEDECINE : I_2_I_0_I_0_I_2_I

N° d'inscription au CONSEIL DE L'ORDRE : **63/5048**

N° ADELI: **631046620**

SPECIALITE : Anesthésie-Réanimation

FONCTIONS ACTUELLES :

- HOPITAL - Praticien Hospitalier (PH)
- UNIVERSITE - Participation à l'enseignement

………………………………………………………………

PARTICIPATION ANTERIEURE A DES RECHERCHES BIOMEDICALES (en particulier dans le domaine de la recherche visée, publications éventuelles) :

-Etudes multicentriques réalisées dans le service :

- HL10 : Effet du surfactant exogène sur la survie des patients en SDRA.

- ACURASYS : comparaison de 2 régimes de curares dans le SDRA.

- DOLOREA : étude de la douleur en réanimation.

- EARSS : étude comparative de l'utilisation de l'albumine dans le sepsis

5 Publications internationales

[1] Constantin JM, Schneider E, Cayot-Constantin S, Guerin R, Bannier F, Futier E, et al. Remifentanil-based sedation to treat noninvasive ventilation failure: a preliminary study. Intensive Care Med. 2007 Jan;33(1):82-7.

[2] Constantin JM, Cayot S, Roszyk L, futier E, Sapin V, Bazin JE, et al. The Response to Recruitment Maneuver Influences Net Alveolar Fluid Clearance in Acute Respiratory Distress Syndrome. Anesthesiology. 2007;

[3] Constantin JM, Jaber S, Futier E, Cayot-Constantin S, Verny-Pic M, Jung B, et al. Respiratory effects of different recruitment maneuvers in acute respiratory distress syndrome. Critical care 2008;12:R50.

DATE : I_3_I_0_I I_0_I_8_I I_2_I_0_I_0_I_7_I SIGNATURE :***S CAYOT-CONSTANTIN***

ANNEXE 6 : Cahier de recueil des données

Echographie pulmonaire et cardiaque couplée au BNP lors du sevrage de la ventilation mécanique

Id Patient (étiquette) :

Date : **Opérateur :**

1 Données démographiques :

Age : **Sexe :** **Poids actuel** **Poids J0 :**

Taille : **traitement habituel :**

Admission :

Polytrauma / Neurochir / Viscéral-uro / Vasculaire / Cardiochir / Maxillo

Medical

Antécédents :

Cardiopathie : oui / non type : Valve / HTA / ischémique

BPCO

SOFA adm **IGS2 admission :**

Lien utile www.sfar.org (onglet Utilitaire / Scores)

Durée de ventilation en jours :

Motif de ventilation/IOT

Cause de non extubation précoce pour les post-opératoires :

2 Variables cliniques :

	VS AI PEP	VS Tube 1 heure	Post extub H4
FC			
PAS/PAD/PAM			
FR			
Spo2			
Débit O2 (L/min)			
Diurèse des 24 heures			
Diurétiques au cours des 24 h précédentes			
Balance hydrique des dernières 24 h			

3 Biologie :

CLEARANCE CREAT : ML/MN

	**VSAI =**<br>**PEP=**<br>**FIO2=**	**VS Tube H1**	**Post extub H4-6**
**PH**			
**PaO2**			
**PaCO2**			
**HCO3-**			
**BNP**			

4 Echographie cardiaque:

	VSAI PEP	VS Tube	Post extub H4-6
FRS			
Cinétique			
CCVG			
ITV sous Ao			
FC			
Vmax IT			
Vélocité Onde E			
TDE			
Vélocité onde A			
DTI Ea latéral			

5 Echo Pulmonaire :

<u>Scores de gain / perte d'aération :</u>

VS/tube vs VSAI =

Extubation H4-6 vs VS/Tube =

Extubation H4-6 vs VSAI =

6 Reventilation avant H48

oui / non

Préciser : (un ou plus)

Œdème laryngé/ stridor

Spo2 < 90% sous FiO2> 50%

Acidose respiratoire pH > 7.30 , Pa CO2 > 50 mmHg

Signes cliniques de détresse respiratoire

Encombrement bronchique / toux inefficace

OAP cardiogénique

Atélectasies

Coma , encéphalopathie

Autres

NB SI BPCO

SI VNI en post extubation chez un patient BPCO, bien noter le nombre d'heures de VNI quotidienne, ainsi que le pH artériel , et le nombre d'heure de VNI à domicile avant le séjour en réanimation le cas échéant

PROTOCOLE PULCO

Opérateur (initiales) :

Etiquette Patient

Secteur :
☐ 1 Rea Gaston Cordier
☐ 2 Rea Husson Mourier
☐ 2 Rea Clermont-Ferrand

Date :

Heure :

COCHER /

☐VS AI **☐ VS/TUBE** **☐ EXTUBATION H4_H6**

DROITE

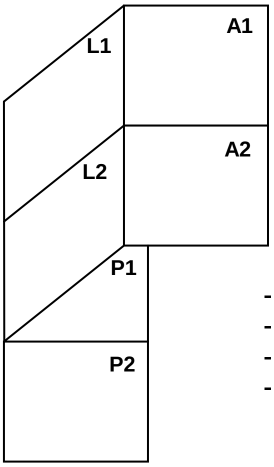

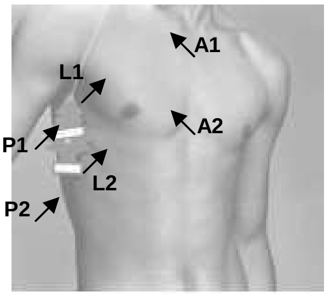

GAUCHE

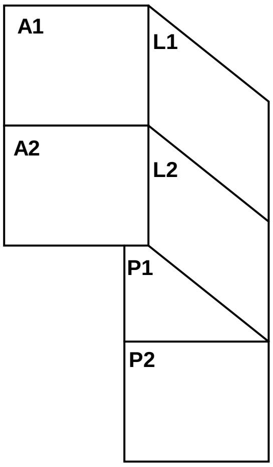

- 0 : Normal
- 1 : Queues de Comètes espacées
- 2 : QC en Rideau
- 3 : Consolidation

LUS = (somme des points) =
