# Supplementary material for: Plasma Levels of sRAGE, Loss of Aeration and Weaning Failure in ICU Patients: A Prospective Observational Multicenter Study
Source: PLoS One. 2013 May 27;8(5):e64083. doi: 10.1371/journal.pone.0064083 (PMC3664630; doi:10.1371/journal.pone.0064083)
Supplement: Protocol S1 — Trial Protocol (english and french versions, protocol amendment, ethics committee approval). (ZIP) [file pone.0064083.s002.zip › Study protocol sRAGE_weaning-PLoS/Pulco-sRAGE_weaning amendment ENG.pdf]

Pulmonary and cardiac ultrasound coupled with BNP measurements during weaning from mechanical ventilation

Amendment to study protocol:

Assessment of pulmonary alveolar epithelium dysfunction during weaning from mechanical ventilation: an ancillary study of plasma levels of sRAGE

Short Title: PULCO_sRAGE
Version 2: 26/01/2010 – Amended
No EudraCT/AFSSAPS: 2009-A01310-57
Promoter code: RBHP 2009 PERBET
ClinicalTrials.gov Identifier: NCT01098773

Sponsor
CHU Clermont-Ferrand
58 Rue de Montalembert
63003 Clermont-Ferrand Cedex 1

Principal Investigator:
Dr. Matthieu JABAUDON
Adult Intensive Care Unit, Anesthesiology & Critical Care Department, Hôtel-Dieu
CHU Clermont-Ferrand
mjabaudon@chu-clermontferrand.fr
Tel: 04-73-750-501

Methodologist:
Bruno Pereira, PhD (Biostatistics)
Department of Clinical Research and Innovation
CHU Clermont-Ferrand
04 73 754 964

Collaborators:
- Intensive Care Unit, Anesthesiology & Critical Care Department, Hôtel-Dieu, CHU Clermont-Ferrand
- Intensive Care Unit, Department of Anesthesiology, AP-HP, Groupe Hospitalier Pitie-Salpetriere

SUMMARY

Weaning from mechanical ventilation is a critical period in intensive care unit (ICU) patients. Weaning failure includes initial spontaneous breathing trial (SBT) failure, postextubation distress and death occuring within 48h following extubation. Postextubation distress is defined as reintubation or need for non-invasive ventilation within 48 hours following extubation. Following a successful SBT, incidence of reintubation ranges between 3 and 30%. Postextubation distress after a successful SBT is associated with increased morbidity and mortality. Given the risks associated with delayed or unsuccessful extubation, determining readiness for extubation and predicting postextubation distress is a critical challenge in the ICU. Most of proposed predictors of postextubation distress either require special equipment, or are too complex for bedside use, or have a limited predictive value. To date, there are no simple clinical indices known to be powerful predictors of postextubation distress. Many mechanisms may impact on the ability to wean from mechanical ventilation, including spontaneous breathing-induced cardiac failure, and neuromuscular disorders, or alteration of lung resistance and compliance. Based on recent findings, a 60-minute SBT is associated with significant lung derecruitment, as assessed by transthoracic lung ultrasound, and among patients who successfully pass SBT, the derecruitment is greater in patients who develop postextubation distress than in those who do not. Factors leading to such a derecruitment have been poorly investigated to date. Among them, alveolar epithelial dysfunction, its repair, and their putative roles in maintaining lung homeostasis could be rather novel and unexplored candidates. As recently shown by our group and other teams, the soluble form of the receptor for advanced glycation end-products (sRAGE) is a marker of alveolar type I epithelial cell injury, and levels of sRAGE are elevated during acute respiratory distress syndrome (ARDS). The association between SBT-induced loss of aeration and alveolar epithelial injury has never been investigated to date, and it remains unknown whether plasma levels of sRAGE could be useful in identifying patients at risk for postextubation distress during the process of weaning from mechanical ventilation.

Therefore, the objectives of this study were to determine whether plasma levels of sRAGE are associated with postextubation distress and/or weaning failure, and to determine whether lung aeration loss during a successful weaning trial could be explained by alveolar epithelium dysfunction, as investigated by sRAGE levels.

Type of study: Biomedical, prospective, observational, open, nonrandomized.

Number of centers:
1. Adult Intensive Care Unit, Prof. Bazin, Hôtel Dieu, CHU Clermont-Ferrand.
2. Intensive care unit, ROUBY Pr, Pr DAR Coriat, AP-HP Pitié-Salpêtrière, Paris

Description of the study:
When available, stored plasma samples will be tested for duplicate measurements of plasma levels of sRAGE, using commercially available ELISA kits (R&D, USA).

Ethics Committee
The "*Comité de Protection des Personnes (CPP) Sud Est VI*" (CHU Clermont-Ferrand, France) is asked to approve this ancillary study (N° AU-823, AFSAPS 2009-A-O1310-57).

As no new blood sampling is planned, the CPP gave its approval on September 25th, 2010.
