# Supplementary figures and images for: Plasma Levels of sRAGE, Loss of Aeration and Weaning Failure in ICU Patients: A Prospective Observational Multicenter Study
Source: PLoS One. 2013 May 27;8(5):e64083. doi: 10.1371/journal.pone.0064083 (PMC3664630; doi:10.1371/journal.pone.0064083)

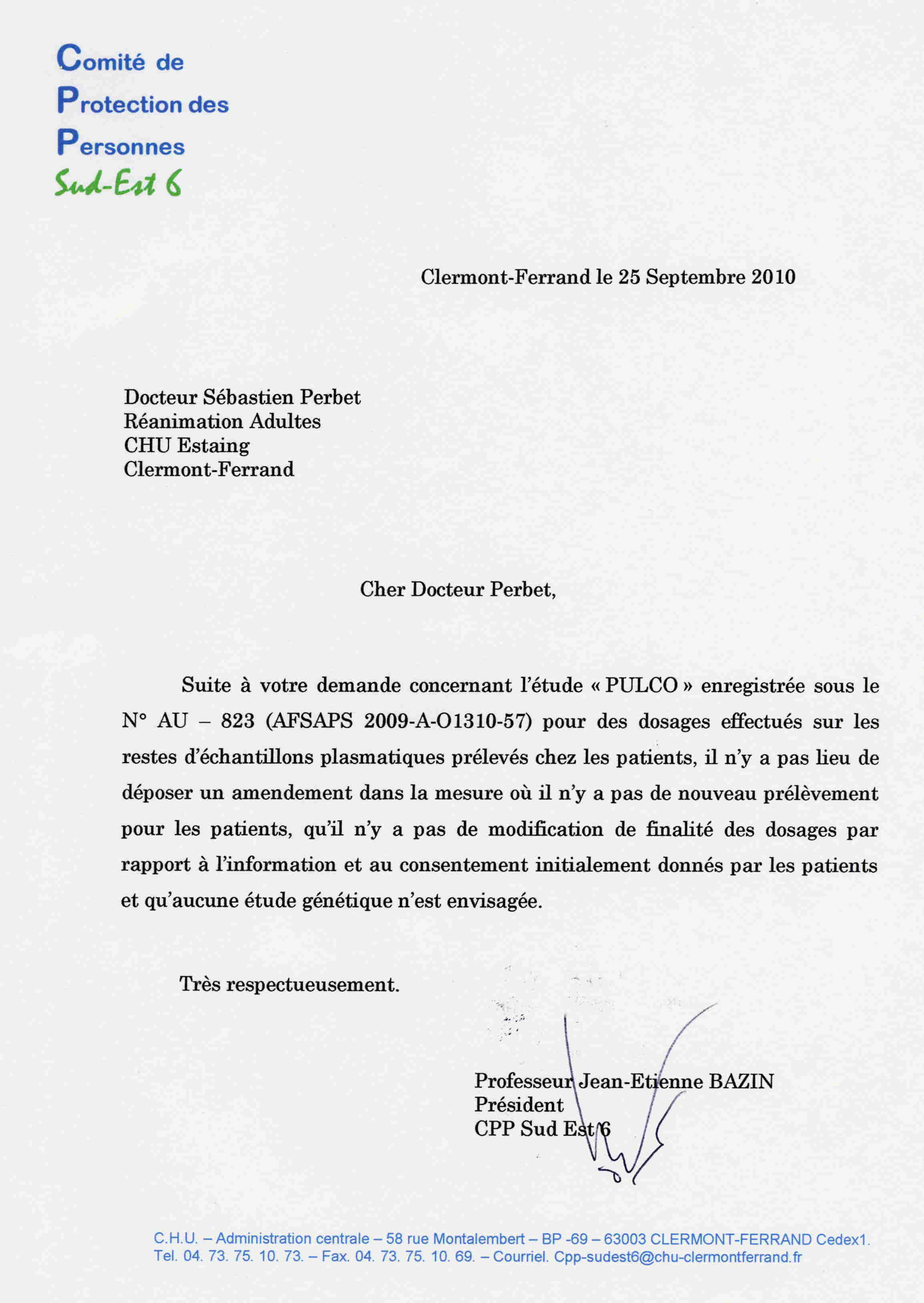

Supplement: Protocol S1 — Trial Protocol (english and french versions, protocol amendment, ethics committee approval). (ZIP) [file pone.0064083.s002.zip › Study protocol sRAGE_weaning-PLoS/Lettre_cpp_Pulco-sRAGE.jpg]
